# Elafin inhibits obesity, hyperglycemia, and liver steatosis in high-fat diet-treated male mice

**DOI:** 10.1038/s41598-020-69634-3

**Published:** 2020-07-30

**Authors:** Jiani Wang, Christina Ortiz, Lindsey Fontenot, Riya Mukhopadhyay, Ying Xie, Ivy Ka Man Law, David Q. Shih, S. Anjani Mattai, Zhaoping Li, Hon Wai Koon

**Affiliations:** 1 Vatche and Tamar Manoukian Division of Digestive Diseases, Department of Medicine, David Geffen School of Medicine, University of California Los Angeles, Room 44-129, Center for Health Sciences Building, 10833 Le Conte Avenue, Los Angeles, CA 90095 USA; 2 Department of Medicine, David Geffen School of Medicine, University of California Los Angeles, Los Angeles, CA 90095 USA; 3 Department of Gastroenterology, First Affiliated Hospital, China Medical University, Shenyang City, Liaoning Province China; 4 Division of Clinical Nutrition, David Geffen School of Medicine, University of California Los Angeles, Los Angeles, CA 90095 USA; 5 F. Widjaja Foundation, Inflammatory Bowel & Immunobiology Research Institute, Cedars-Sinai Medical Center, Los Angeles, CA 90048 USA

**Keywords:** Endocrine system and metabolic diseases, Metabolism

## Abstract

Elafin is an antimicrobial and anti-inflammatory protein. We hypothesize that elafin expression correlates with diabetes. Among non-diabetic and prediabetic groups, men have significantly higher serum elafin levels than women. Men with type 2 diabetes mellitus (T2DM) have significantly lower serum elafin levels than men without T2DM. Serum elafin levels are inversely correlated with fasting blood glucose and hemoglobin A1c levels in men with T2DM, but not women with T2DM. Lentiviral elafin overexpression inhibited obesity, hyperglycemia, and liver steatosis in high-fat diet (HFD)-treated male mice. Elafin-overexpressing HFD-treated male mice had increased serum leptin levels, and serum exosomal miR181b-5p and miR219-5p expression. Transplantation of splenocytes and serum exosomes from elafin-overexpressing HFD-treated donor mice reduced food consumption and fat mass, and increased adipose tissue leptin mRNA expression in HFD-treated recipient mice. Elafin improved leptin sensitivity via reduced interferon-gamma expression and induced adipose leptin expression via increased miR181b-5p and miR219-5p expression. Subcutaneous and oral administration of modified elafin inhibited obesity, hyperglycemia, and liver steatosis in the HFD-treated mice. Circulating elafin levels are associated with hyperglycemia in men with T2DM. Elafin, via immune-derived miRNAs and cytokine, activates leptin sensitivity and expression that subsequently inhibit food consumption, obesity, hyperglycemia, and liver steatosis in HFD-treated male mice.

## Introduction

The Centers for Disease Control and Prevention (CDC) reported that 8.6% of U.S. adults are diagnosed with type 2 diabetes mellitus (T2DM)^1^. T2DM is characterized by hyperglycemia with a combination of insulin resistance and relative insulin deficiency. Globally, more men are diagnosed with T2DM than women^2,3^. The development of T2DM involves many factors and is a topic of intense research^4^.

Recent reports suggest the relevance of antimicrobial peptides (cathelicidin and lactoferrin) in the regulation of diabetes^5–7^. Interestingly, one of the antimicrobial peptides, elafin, is abundantly expressed in the urinary extracellular vesicles in patients with type I diabetes mellitus (T1DM)^8^. Its level is progressively decreased when these patients develop diabetic nephropathy, suggesting its involvement in diabetes. Elafin is a small (6 kDa) human elastase-specific protease inhibitor and antimicrobial peptide primarily expressed in immune cells, intestinal tract, vagina, lungs, and skin^9,10^. Circulating elafin levels are positively correlated with inflammatory bowel disease and graft-versus-host disease^10,11^. Elafin expression is also increased in human atherosclerotic coronary arteries and mesenteric fat in stricturing Crohn’s disease patients^10,12^. However, the circulating levels of elafin in patients with T2DM are unknown.

Elafin possesses anti-inflammatory effects as elafin inhibits lipopolysaccharide (LPS)-mediated inflammatory responses including activator protein 1 (AP-1) and Nuclear Factor kappa-light-chain-enhancer of activated B cells (NF-κB) in monocytes^13^. Elafin also reduces interleukin-8 (IL-8) production in endothelial cells exposed to oxidized low-density lipoprotein (oxLDL)^14^. In utilizing elafin for therapeutic applications, subcutaneous injections of elafin can reverse pulmonary hypertension in rats^15^. Additionally, oral administration of elafin-expressing *Lactococcus* ameliorates dextran sulfate (DSS)- and trinitrobenzene sulfonic acid (TNBS)-mediated colitis in mice and gluten-related disorders in humans^16,17^. However, the therapeutic potential of elafin in diabetes is unknown.

As antimicrobial peptides are associated with diabetes, we hypothesize that a link between elafin expression and diabetes may exist. Our study included a cohort of patients for determining the serum elafin levels in non-diabetic, prediabetic, and diabetic (T2DM) patients. This study discovered the unique clinical significance of abnormal elafin expression in patients with T2DM. We utilized well established high-fat diet (HFD)-treated mice as diet-induced obesity (DIO) model for T2DM^18^. Through the application of B- and T-cell deficient *Rag*^−/−^ mice, splenocyte transplantation, and serum exosome transplantation, this study is also the first to elucidate the mechanistic connection between cytokine, miRNA, and hormone in the elafin-mediated regulation of obesity, hyperglycemia, and liver steatosis in HFD-treated mice. We developed two clinically useful elafin delivery approaches and evaluated their anti-diabetic efficacies in HFD-treated mice.

## Results

### Circulating elafin levels are inversely correlated with fasting blood glucose and HbA1c levels in men with T2DM

**Figure 1:**
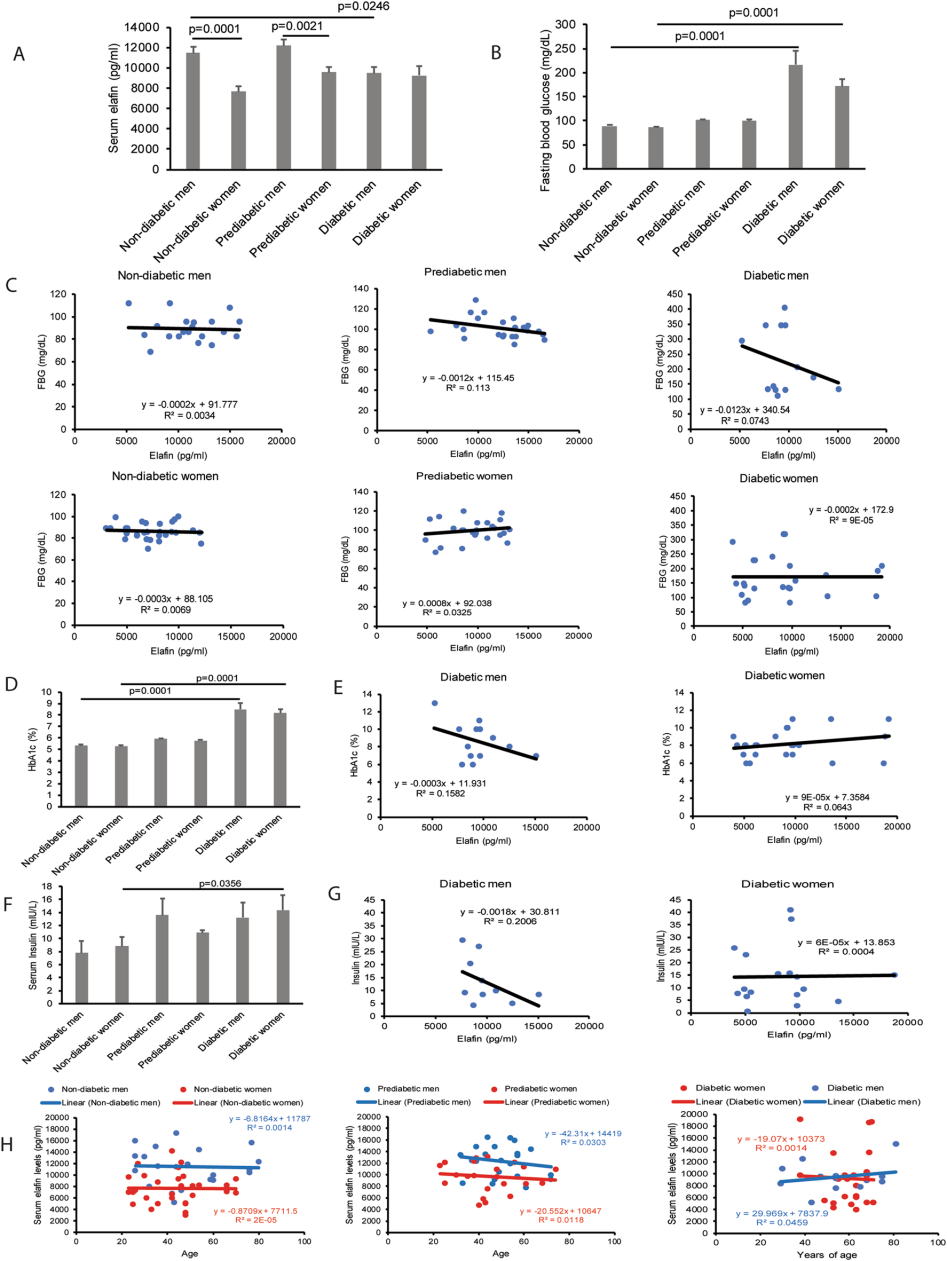
Serum elafin levels were inversely correlated with hyperglycemia and hyperinsulinemia in men with T2DM. (**A**, **B**) Serum elafin and fasting blood glucose levels in patients. (**C**) The inverse correlation between serum elafin levels and fasting blood glucose levels in men with T2DM. No correlation between elafin and fasting blood glucose levels in patients without prediabetes/diabetes, patients with prediabetes, and women with T2DM. (**D**) HbA1c levels in patients. (**E**) The inverse correlation between serum elafin levels and HbA1c levels in men with T2DM but not women with T2DM. (**F**) Fasting blood insulin levels in patients. (**G**) The inverse correlation between serum elafin levels and fasting blood insulin levels in men with T2DM but not women with T2DM. (**H**) There is no association between serum elafin levels and age among all patients.

To determine the association between circulating elafin protein and diabetes, we measured serum elafin levels of 53 patients without prediabetes/diabetes, 48 patients with prediabetes, and 38 patients with (T2DM). The baseline characteristics of this cohort are shown in Table S1. Among non-diabetic and prediabetic groups, men have significantly higher circulating elafin levels than women (Fig. 1A). Men with T2DM have significantly lower circulating elafin levels than men without diabetes (Fig. 1A). Women with T2DM have relatively low circulating elafin levels, similar to women without prediabetes/diabetes and women with prediabetes (Fig. 1A). All patients with T2DM have significantly higher fasting blood glucose and hemoglobin A1c (HbA1c) levels than non-diabetic and prediabetic patients (Fig. 1B,D).

Women with T2DM also have significantly higher fasting blood insulin levels than women without prediabetes/diabetes (Fig. 1F). Men with T2DM and prediabetes also have higher blood insulin levels than men without diabetes, but the differences were statistically insignificant (Fig. 1F). Serum elafin levels are inversely correlated with fasting blood glucose, HbA1c, and insulin levels in men with T2DM, but not women with T2DM (Fig. 1C,E,G). However, circulating elafin levels are independent of age or body mass index (BMI) of all patients (Fig. 1H and S1A–B). These findings suggest that elafin may reduce the severity of diabetes in men, leading us to further pursue this direction with animal models.

### Lentiviral elafin overexpression reduced high-fat diet-induced obesity, hyperglycemia, and hyperphagia in male mice

**Figure 2:**
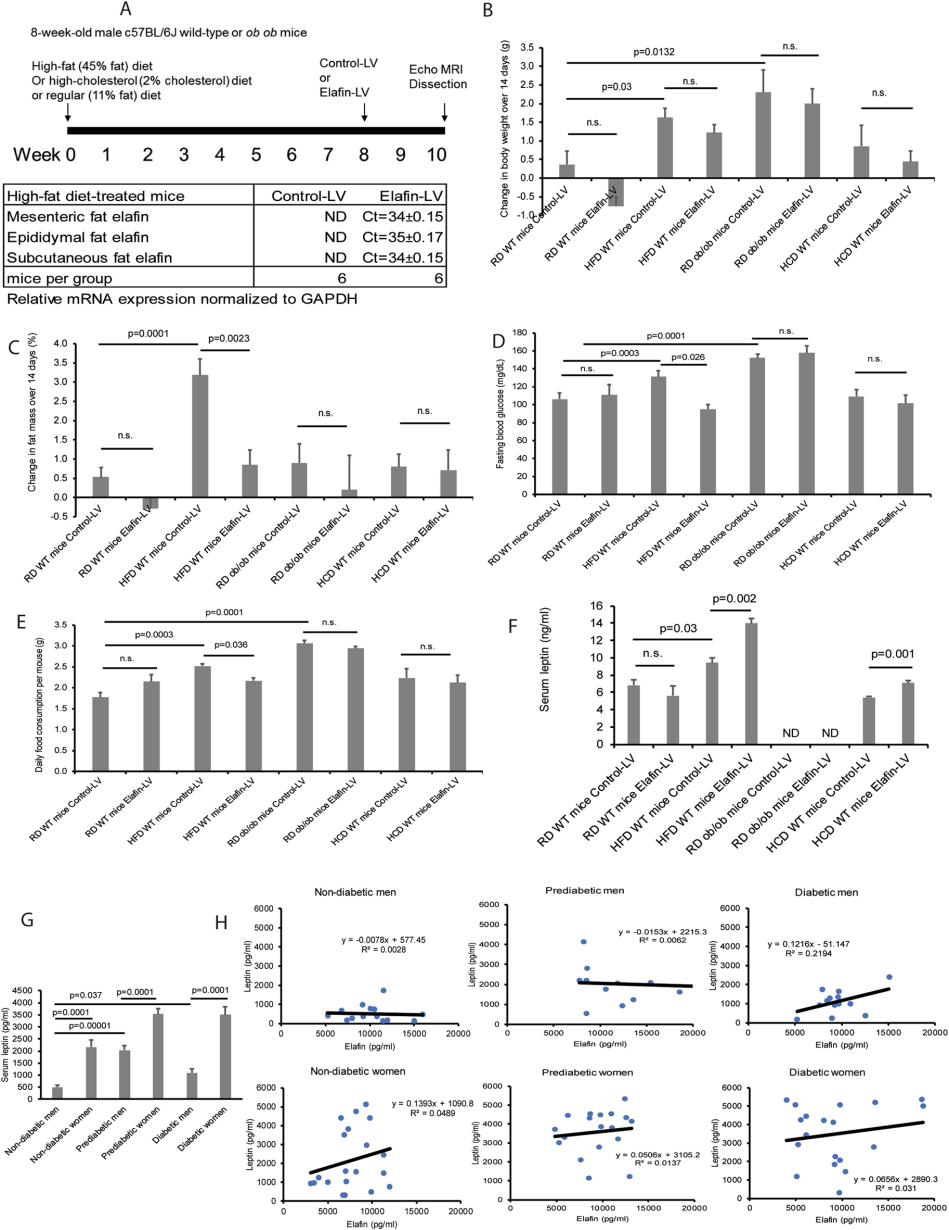
Lentiviral elafin overexpression reduced food consumption and fat mass in HFD-treated male mice. (**A**, upper panel) RD/HFD/HCD treatment and lentiviral elafin overexpression in mice. (**A**, lower panel) Ct values in the real-time RT-PCR experiments for detecting the presence of elafin mRNA signal in the adipose tissues of HFD-treated mice. Each group consists of 6 mice. (**B**) Change in body weight over 14 days. (**C**) Change in percentage of fat mass. (**D**) Fasting blood glucose levels. (**E**) Daily food consumption. (**F**) Serum leptin levels. Elafin did not affect food consumption, fat mass, or fasting blood glucose levels in *ob/ob* mice and HCD-treated mice. (**G**) Serum leptin levels in patients. Women have significantly higher serum leptin levels than men in all groups. (**H**) The correlation between serum elafin levels and fasting blood glucose levels in patients. Serum elafin levels are positively correlated with leptin levels in men with T2DM.

To evaluate the physiological effects of elafin in diet-induced obesity, hyperglycemia, or hypercholesterolemia, 8-week-old male c57BL/6J wild-type mice received 8-week regular diet (RD), high-fat diet (HFD), or low-fat high-cholesterol diet (HCD) treatment, followed by intravenous injection with either control lentivirus (control-LV) or elafin-expressing lentivirus (elafin-LV)^5^. Measurements of the various parameters were performed 2 weeks after lentiviral infection (Fig. 2A, upper panel). Mice do not have an elafin gene; therefore, the elafin mRNA signal was undetectable in mice infected with control-LV (Fig. 2A, lower panel). However, the elafin mRNA signal was positive in adipose tissues of elafin-overexpressing mice (elafin-LV group) only (Fig. 2A, lower panel). Elafin protein was detected in the sera of elafin-expressing mice only, i.e., 0.28 ± 0.03 ng/ml (mean ± sem) (Fig. 6B).

RD-treated male mice had normal fasting blood glucose levels (106 ± 7 mg/dL), which were comparable to the findings of other studies^19,20^. The elafin-mediated reduction of body weight gain and fat mass gain in RD-treated mice was statistically insignificant (Fig. 2B,C). Elafin overexpression did not affect food consumption, fasting blood glucose levels, and serum leptin (appetite-controlling hormone) levels in the RD-treated male mice (Fig. 2D–F).

HFD-treated male mice displayed prediabetic phenotypes with significantly higher body weight, fat mass, fasting blood glucose levels, and food consumption than RD-treated male mice (Fig. 2B–E)^5^. The fasting blood glucose (FBG) levels in our HFD-treated male mice were 131 ± 7 mg/dL, which is regarded as prediabetic^21^. As this study sought to determine the therapeutic effects of elafin against diabetes, female mice were not included because HFD-treated female mice do not develop hyperglycemia^22^. Elafin overexpression significantly reduced fat mass gain (by 2.3%), fasting blood glucose levels (by 27%), and food consumption (by 13.8%) in HFD-treated, but not in HCD-treated male mice within 14 days (Fig. 2C–E). In an oral glucose tolerance test (OGTT), glucose feeding elevated blood glucose levels in RD- and HFD-treated mice (Figure S2A)^5^. Elafin overexpression modestly reduced blood glucose levels in the HFD-treated mice, but the difference was statistically insignificant (Figure S2A).

Consistent with previous studies^23–25^, HFD treatment increased circulating insulin and total cholesterol, but not free fatty acid and adiponectin levels in mice (Figure S2B–E). HCD treatment increased circulating total cholesterol levels without affecting fat mass and fasting blood glucose levels in mice (Figure S2C, Fig. 2C–D). Elafin overexpression did not significantly affect body weight, insulin, total cholesterol, free fatty acid, and adiponectin levels in the HFD-treated and HCD-treated mice (Fig. 2B, Figure S2B–E).

### Elafin reduced food consumption via increased leptin expression in mesenteric fat of HFD-treated male mice

To explain the decreased food consumption in elafin-overexpressing mice, we measured leptin levels in blood and leptin mRNA expression in fat tissues as leptin, an adipose-derived hormone, reduces food intake^26^. HFD-treated mice, but not HCD-treated mice, have significantly higher circulating leptin levels than RD-treated mice (Fig. 2F). Elafin-overexpressing mice had higher circulating leptin levels than control lentivirus-expressing mice in both HFD and HCD treatment (Fig. 2F). Leptin was not detectable in the sera of *ob/ob* mice as they are leptin-deficient (Fig. 2F). We included leptin-deficient *ob/ob* male mice to confirm the role of leptin in this phenotype. The *ob/ob* mice had significantly higher body weight gain, fasting blood glucose levels, and food consumption than wild-type mice, which were not affected by elafin overexpression (Fig. 2B–E).

Similarly, patients with prediabetes and T2DM have significantly higher circulating leptin levels than patients without diabetes (Fig. 2G). Women also have significantly higher circulating leptin levels than men among all groups (Fig. 2G). Circulating leptin levels are positively correlated with circulating elafin levels in men with T2DM, but not in women with T2DM (Fig. 2H).

The HFD treatment increased long-chain fatty acid transporter and scavenger receptor Cd36 and leptin mRNA expression in mesenteric and epididymal fat in mice, compared to regular diet treatment (Figure S2E). Elafin overexpression significantly increased leptin mRNA expression in mesenteric fat only (Figure S3A) and decreased fat receptor Cd36 mRNA expression in both mesenteric and epididymal fat in the HFD-treated mice (Figure S3A).

We considered the possible involvement of intestinal microbiota in the actions of elafin. Cecal microbiota transplantation from the elafin-overexpressing donor mice did not significantly affect the fat mass, body weight, fasting blood glucose levels, and food consumption in the HFD-treated recipient mice (Figure S3B). Elafin overexpression does not appear to affect the intestinal environment.

### Immune cells mediate the protective effects of elafin in HFD-treated Rag^−/−^* male mice*

Since elafin is an anti-inflammatory protein^14^^,^ we utilized multiplex ELISA to profile serum cytokines of patients and evaluated the association between circulating elafin and inflammation in patients (Figure S4A). Serum IFNγ levels are inversely correlated with serum elafin levels in men with T2DM, but not women with T2DM (Figure S4C–D). Serum IL-1β levels are not associated with serum elafin levels in patients with T2DM (Figure S4A).

**Figure 3:**
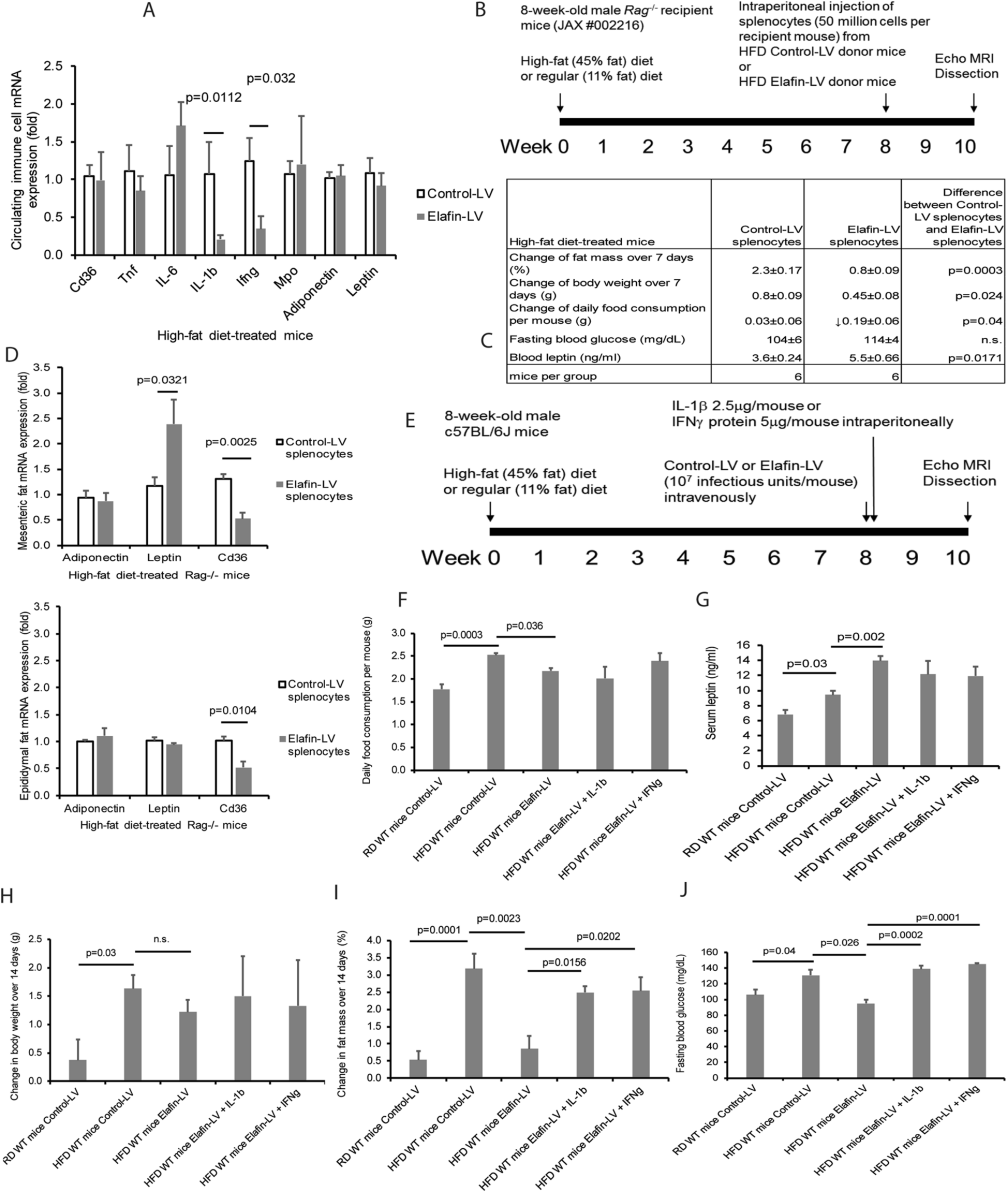
Circulating immune cells mediated the anti-obesity effects of elafin in HFD-treated male mice. (**A**) Circulating immune cell mRNA expression in the HFD-treated mice with and without lentiviral elafin overexpression. (**B**) Splenocyte transplantation to HFD-treated *Rag*^−/−^ mice. (**C**) Physiological parameters of HFD-treated *Rag*^−/−^ recipient mice after splenocyte transplantation. Transplantation of splenocytes from elafin-overexpressing donors caused reduced fat mass, body weight, and food consumption and increased leptin levels in the *Rag*^−/−^ recipient mice. (**D**) Mesenteric fat and epididymal fat tissue mRNA expression in HFD-treated mice with and without lentiviral elafin overexpression. (**E**) Determination of the physiological effects of intraperitoneal IL-1β and IFNγ injection to HFD-treated elafin-expressing mice. IL-1β or IFNγ was injected on the same day as elafin-expressing lentivirus injection. (**F**) Daily food consumption, (**G**) serum leptin levels. (**H**) Change in body weight. (**I**) Change in fat mass. (**J**) Fasting blood glucose levels. Each group consists of 8 mice.

We evaluated the cytokine and hormone mRNA expression in circulating white blood cells. HFD-treated control-LV and elafin-LV mice had similar myeloperoxidase/Mpo (neutrophil), and adiponectin and leptin (hormone) mRNA expression in their circulating white blood cells (Fig. 3A). Interestingly, elafin overexpression significantly reduced interleukin-1beta (IL-1β) and interferon-gamma (IFNγ), but not tumor necrosis factor (Tnf) and interleukin-6 (Il-6), mRNA expression in circulating white blood cells of the HFD-treated mice (Fig. 3A), indicating the involvement of circulating immune cells and cytokines in the elafin-mediated anti-diabetic effects.

We transplanted splenocytes from HFD-treated control-LV or elafin-LV mice to HFD-treated *Rag*^−/−^ recipient mice, which are mature B and T lymphocytes deficient (Fig. 3B)^27^. This approach enabled us to investigate the effects of elafin-conditioned immune cells without using direct elafin overexpression in the HFD-treated recipient mice. After the injection of splenocytes from HFD-treated control-LV male donor mice, the HFD-treated *Rag*^−/−^ male recipient mice continued to increase fat mass and body weight (Fig. 3C). Recipient mice of HFD-treated elafin-overexpressing mouse (Elafin-LV) splenocytes showed significantly increased serum leptin levels with decreased fat mass gain (by 1.5%), body weight gain (by 44%), and food consumption (by 0.19 g), compared to those injected with control splenocytes (Fig. 3C). Fasting blood glucose levels were not significantly affected by splenocyte transplantation (Fig. 3C) because the 129 strain recipient mice were resistant to the development of insulin resistance^28^. Similar to the elafin-overexpressing donor mice, recipient mice of elafin-overexpressing mouse splenocytes had increased leptin mRNA expression in mesenteric fat only and reduced Cd36 mRNA expression in the mesenteric fat and epididymal fat (Fig. 3D).

### IFNγ is involved in the elafin-dependent regulation of food consumption in HFD-treated male mice

To determine the involvement of immune cell-derived IL-1β and IFNγ in the elafin-mediated effects, we injected IL-1β and IFNγ protein to the HFD-treated elafin-overexpressing mice intraperitoneally (Fig. 3E). IFNγ, but not IL-1β, moderately increased food consumption in the elafin-overexpressing mice, suggesting that elafin-dependent IFNγ expression may affect leptin sensitivity (Fig. 3F). Injection of either cytokine reversed the elafin-mediated inhibition of fat mass and fasting blood glucose levels, but not serum leptin levels and body weight gain of the HFD-treated elafin-overexpressing mice (Fig. 3G–J).

### Circulating exosomes and immune cells shared similar miRNA expression profiles in elafin-overexpressing HFD-treated male mice

**Figure 4:**
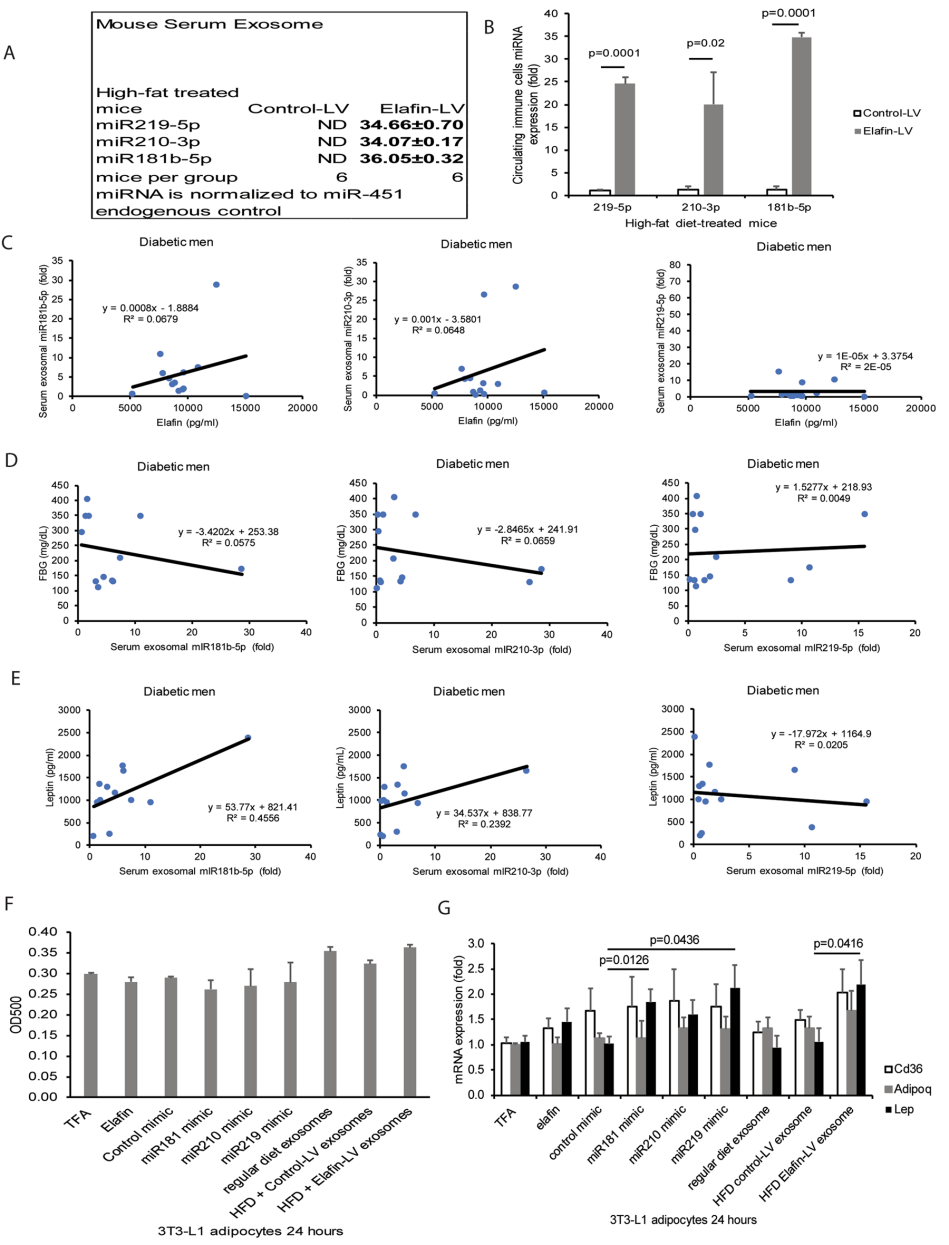
Immune cell-derived miR181b-5p and miR219-5p induced leptin mRNA expression in 3T3-L1 adipocytes. (**A**) Mouse serum exosomal miRNA expression was profiled by a PCR array. miR219-5p, miR210-3p, and miR181-5p were undetectable in the serum exosomes of HFD-treated mice without elafin overexpression. These three miRNAs were detected in the serum exosomes of elafin-overexpressing HFD-treated mice only. Each group consists of 6 mice. (**B**) miR219-5p, miR210-3p, and miR181-5p expression in the circulating immune cells were significantly increased in the elafin-overexpressing HFD-treated mice, compared to HFD-treated mice without elafin overexpression. (**C**) The positive correlation between serum elafin levels and miRNAs (miR181b-5p and miR210-3p, but not miR219-5p) in the men with T2DM. (**D**) The negative correlation between fasting blood glucose levels and miRNAs (miR181b-5p and miR210-3p, but not miR219-5p) in men with T2DM. (**E**) The positive correlation between serum leptin levels and miRNAs (miR181b-5p and miR210-3p, but not miR219-5p) in the men with T2DM. (**F**) Oil red O staining of serum-starved 3T3-L1 adipocytes after 24-h exposure to elafin (10 ng/ml, no transfection), miRNA mimics (75 ng/ml via overnight transfection), or mouse serum exosomes (10 µg/ml). (**G**) Leptin, Cd36, and adiponectin mRNA expression in 3T3-L1 adipocytes after 24-h exposure to elafin (10 ng/ml, no transfection), miRNA mimics (75 ng/ml via overnight transfection), and serum exosomes (10 µg/ml). Results were pooled from three independent experiments.

In addition to cytokine expression, immune cells also regulate immune responses via exosome secretion^29,30^. Exosomes are crucial in disease processes of diabetes by carrying miRNAs to target organs^31–34^. To determine whether elafin influences circulating exosomal miRNA expression, we used a PCR array to profile miRNAs in mouse serum exosomes. Signals of miR219-5p, miR210-3p, and miR181b-5p were detectable in the serum exosomes of HFD-treated, elafin-overexpressing mice only (Fig. 4A). Similarly, elafin overexpression significantly increased miR181b-5p, miR210-3p, and miR219-5p expression in circulating white blood cells, but not in mesenteric and epididymal fat tissues of HFD-treated male mice (Fig. 4B and S3C), suggesting that these three serum exosomal miRNAs are derived from circulating immune cells.

### Circulating exosomal miR181b-5p and miR219-5p expression was correlated with blood glucose and leptin levels in men with T2DM

The serum exosomal miR181b-5p, miR210-3p, and miR219-5p expression in patients are shown in Figure S1C, S1E, and S1G. The associations between serum exosomal miR181-5p, miR210-3p, and miR219-5p expression and circulating elafin levels in patients are shown in Figure S1D, S1F, and S1H.

Interestingly, serum exosomal miR181b-5p and miR210-3p, but not miR219-5p, expression were positively correlated with circulating elafin levels in men with T2DM (Fig. 4C). In these men with T2DM, serum exosomal miR181b-5p and miR210-3p expression were negatively correlated with fasting blood glucose levels but positively correlated with circulating leptin levels (Fig. 4D–E). Circulating exosomal miR219-5p expression was not associated with blood glucose and leptin levels in men with T2DM (Fig. 4D–E).

### Elafin-dependent serum exosomal miR181b-5p and miR219-5p induced leptin mRNA expression in adipocytes

Differentiation of preadipocytes to adipocytes is characteristic of lipid accumulation, adiponectin (adipokine) production, and increased CD36 expression^35–37^. We incubated mouse adipocytes with elafin, miRNA mimics, and exosomes. Exposure to elafin protein, mouse serum exosomes (from all groups), and miRNA mimics (181-5p, 219-5p, and 210-3p) affected neither lipid accumulation (Fig. 4F) nor Cd36 and adiponectin mRNA expression (Fig. 4G) in adipocytes, suggesting that elafin does not affect adipocyte differentiation in vitro.

Serum exosomes from elafin-overexpressing mice, miR181b-5p mimic, and miR219-5p mimic, but not miR210-3p mimic and elafin protein, significantly increased leptin mRNA expression in mouse 3T3-L1 adipocytes (Fig. 4G). Similarly, transfection of either miR181-5p mimic or miR219-5p mimic significantly increased leptin secretion in primary human mesenteric fat adipocytes (Figure S3D). Elafin, via serum exosomal miR181b-5p and miR219-5p, induces leptin expression in adipocytes indirectly. Therefore, we selected miR181b-5p and miR219-5p for functional validation in vivo.

### Elafin mediates anti-obesity and anti-diabetic effects via serum exosomal miR181b-5p and miR219-5p in the HFD-treated male mice

**Figure 5:**
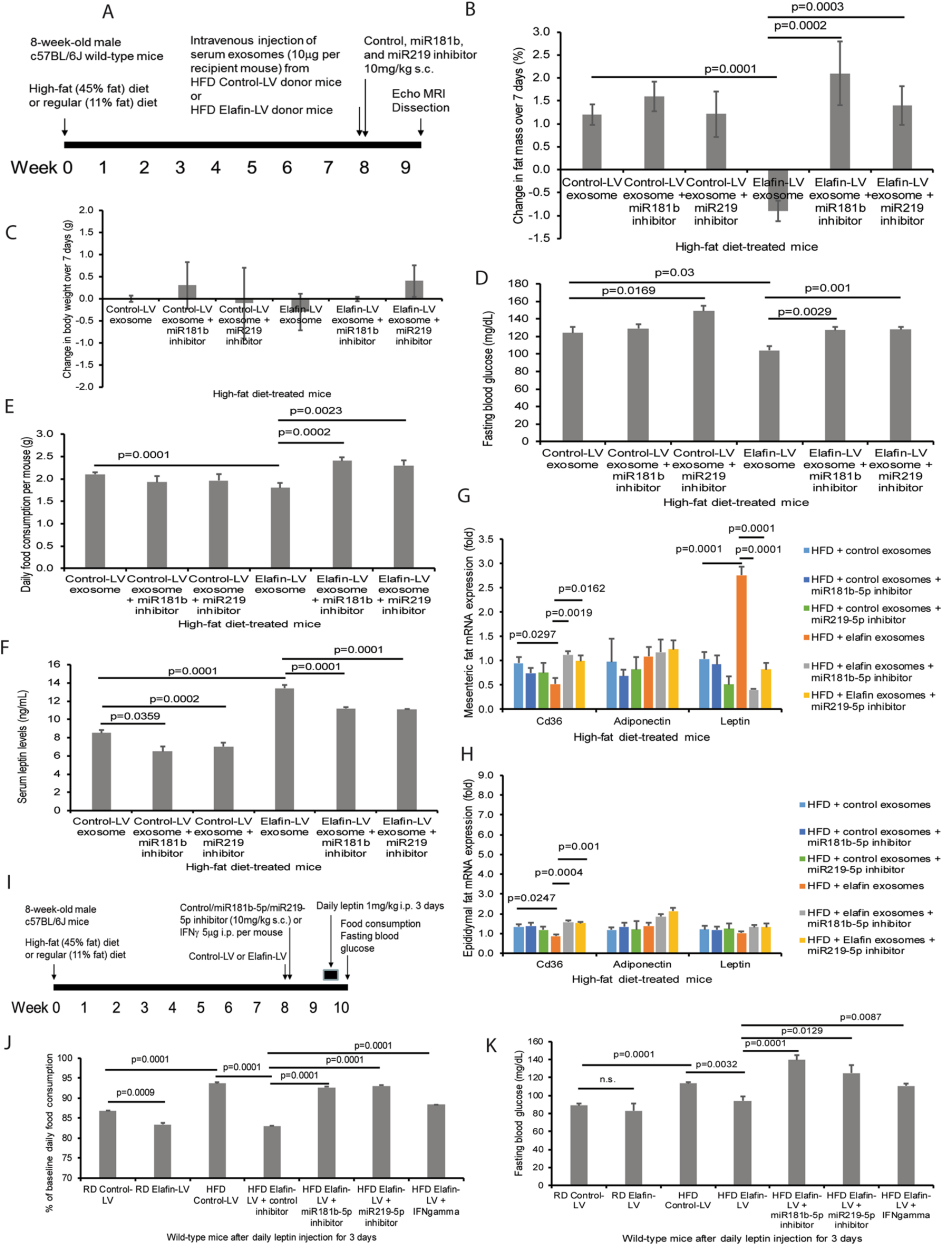
Elafin-dependent serum exosome-mediated inhibition of hyperphagia and hyperglycemia was reversed by miR181b-5p and miR219-5p inhibitors. (**A**) Serum exosome transplantation to HFD-treated mice. The miRNA inhibitors were injected on the same day as exosome injection. (**B**) Change in fat mass. (**C**) No significant change in body weight among all groups over 7 days after miRNA inhibitor and exosome injection. (**D**) Fasting blood glucose levels 7 days after exosome and miRNA inhibitor injection. (**E**) Daily food consumption per mouse. (**F**) Serum leptin levels measured on day seven post-injection. (**G**, **H**) Leptin, Cd36, and adiponectin mRNA expression in the mesenteric fat and epididymal fat of HFD-treated mice with exosome and miRNA inhibitor injection. (**I**) Leptin sensitivity test: The RD/HFD-treated mice were injected with leptin for 3 days after elafin-LV injection. (**J**, **K**) Daily food consumption and fasting blood glucose levels at 24 h after the last leptin injection. Each group consists of 8 mice.

To determine the role of elafin-conditioned circulating exosomes in regulating food consumption, obesity, and hyperglycemia, we transplanted serum exosomes to HFD-treated male recipient mice intravenously (Fig. 5A). Injection of serum exosomes did not significantly affect the body weight in the recipient mice (Fig. 5B). Recipient mice of elafin-overexpressing mouse exosomes had significantly reduced fat mass gain (by 2.1%), fasting blood glucose levels (by 16%), and food consumption (by 14%), compared to those injected with control exosomes (Fig. 5C–E). Injection of elafin-LV serum exosomes did not alter circulating levels of total cholesterol, insulin, free fatty acid, and adiponectin in the recipient mice (Figure S2A–D).

Transplantation of elafin-LV serum exosomes significantly increased serum leptin levels and mesenteric fat leptin mRNA expression in the recipient mice (Fig. 5F–G). The same recipient group also had reduced Cd36 mRNA expression in epididymal and mesenteric fat (Fig. 5G,H). All of these elafin-dependent effects were reversed by miR181b-5p and miR219-5p inhibitors (Fig. 5F–H). Therefore, elafin induces leptin expression and reduces food consumption, obesity, and hyperglycemia via serum exosomal miR181b-5p and miR219-5p.

### Elafin reduces circulating IFNγ levels via serum exosomal miR181b-5p in HFD-treated male mice

In addition, we determined whether elafin-conditioned exosomes affect the circulating levels of cytokines in the HFD-treated mice. HFD treatment significantly increased circulating levels of IFNγ in the mice, which were reduced by elafin-LV serum exosome treatment (Figure S4E). Interestingly, miR181b-5p inhibitor, but not miR219-5p inhibitor, reversed the elafin-dependent suppression of IFNγ levels (Figure S4E). Neither HFD nor elafin-LV treatment affected serum IL-1β levels in the mice (Figure S4E). HFD-treatment also mildly increased (statistically insignificant) the circulating levels of other detected cytokines (such as IL-1β, GM-CSF, IL-12p70, IL-2, IL-3, IL-4, IL-17A, MIP-1α, MIP-1β, and TNFα), but the levels of these ten cytokines were not affected by elafin-LV exosome treatment (Figure S4B). Thus, elafin-dependent exosomes reduced circulating IFNγ levels via miR181b-5p.

### Elafin enhanced leptin sensitivity in the HFD-treated male mice

In addition to elafin-mediated leptin expression, we determined whether elafin affects leptin sensitivity in mice. The HFD-treated male mice received daily intraperitoneal leptin injection for 3 days, followed by measurement of food consumption and fasting blood glucose levels (Fig. 5I). After 3 days of leptin injection, RD-treated elafin-LV group showed moderately reduced food consumption, but not fasting blood glucose levels (Fig. 5J,K). HFD-treated mice showed impaired leptin sensitivity as represented by high food consumption and fasting blood glucose levels after leptin treatment (Fig. 5J,K). Leptin injection further reduced food consumption in HFD-treated elafin-overexpressing mice (Fig. 5J). Fasting blood glucose was reduced in HFD-treated elafin-overexpressing mice, but this decrease was not affected by leptin injection (Fig. 5K). Elafin-mediated decrease of food consumption and fasting blood glucose levels were reversed by subcutaneous injection of miR181b-5p inhibitor, miR219-5p inhibitor, and IFNγ (Fig. 5J,K). Therefore, elafin restores leptin sensitivity in the HFD-treated male mice via increased expression of miR181b-5p and miR219-5p and inhibition of IFNγ.

### Elafin inhibited liver steatosis in the HFD-treated male mice

HFD treatment caused liver steatosis in mice (Figure S5A). Elafin overexpression did not affect the normal liver histology in RD-treated male mice (Figure S5A and B). Elafin overexpression inhibited liver steatosis in the HFD-treated male mice (Figure S5A and B)^5,38^, which was reversed by injection of IL-1β or IFNγ protein (Figure S5A and B). Similar to elafin overexpression, transplantation of splenocytes from elafin-overexpressing mice or circulating exosomes from elafin-overexpressing mice also inhibited liver steatosis (Figure S5C–F). The quantitative changes of steatosis were reflected by non-alcoholic fatty liver steatosis subscore (Figure S5B, D, and F).

HFD treatment significantly increased fat receptor Cd36 mRNA expression in the liver, which was inhibited by either lentiviral elafin overexpression, transplantation of splenocytes from elafin-overexpressing mice, or transplantation of circulating exosomes from elafin-overexpressing mice (Figure S6A–C). The elafin-dependent exosomal inhibition of steatosis and hepatic Cd36 mRNA expression was reversed by miR181b-5p and miR219-5p inhibitors (Figure S5E–F and S6C).

RD-treated leptin-deficient *ob/ob* male mice and HCD-treated male mice developed liver steatosis (Figure S6D–E)^39,40^. Elafin overexpression was ineffective in inhibiting liver steatosis in *ob/ob* mice (Figure S6D). The results suggest that elafin inhibits liver steatosis via leptin expression. Elafin overexpression also failed to inhibit liver steatosis and hepatic Cd36 mRNA expression in the HCD-treated mice (Figure S6A and S6E).

### Subcutaneous PEG-Elafin and oral Elafin-Eudragit formulation inhibited obesity, hyperglycemia, and liver steatosis in the HFD-treated male mice

**Figure 6:**
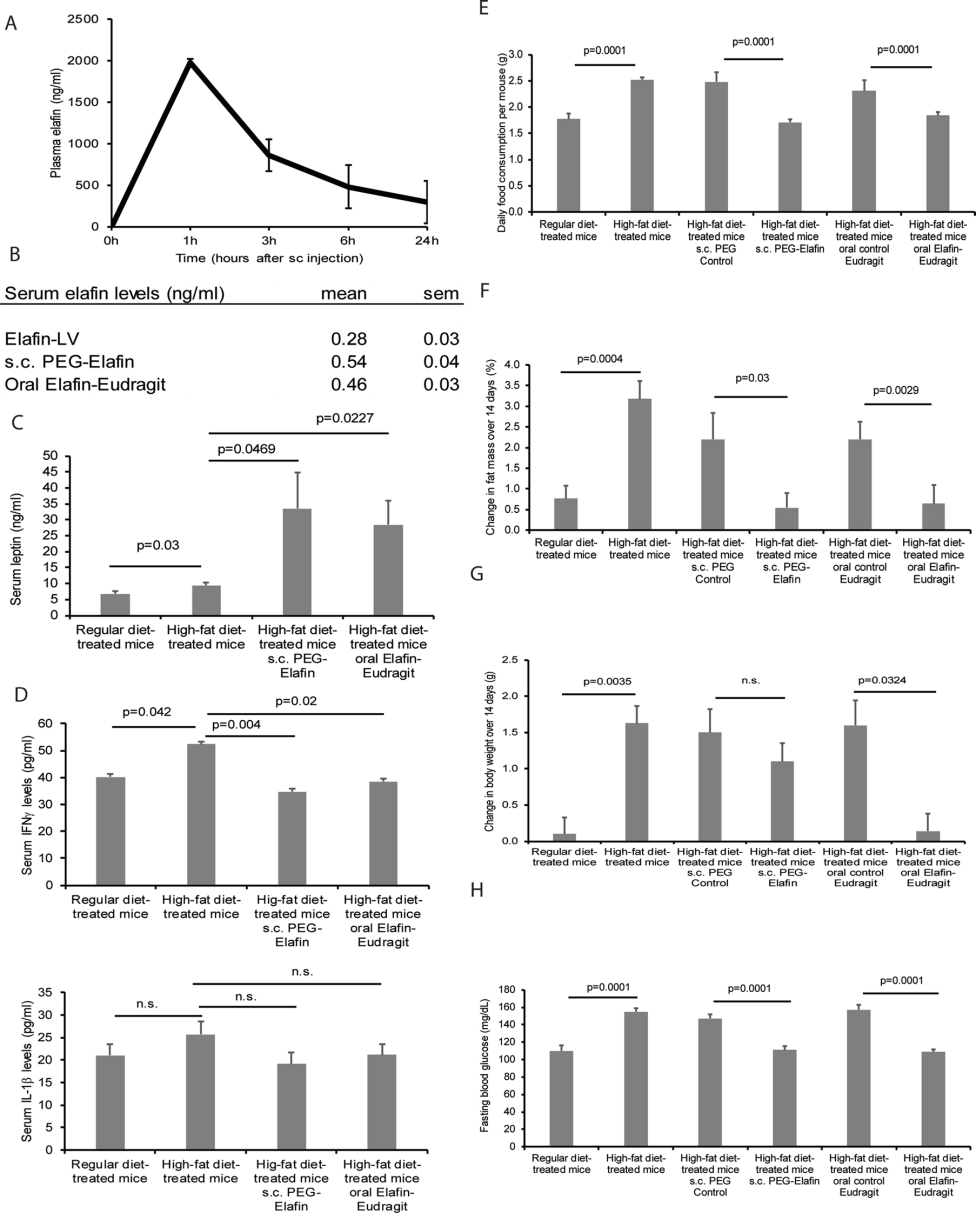
Subcutaneous and oral administration of modified elafin inhibited hyperphagia and hyperglycemia in HFD-treated male mice. (**A**) Elafin (1 mg/kg) was injected into RD-treated male mice subcutaneously. Tail-vein blood samples were collected for elafin ELISA. This mouse experiment was intended for the determination of basic pharmacokinetics of elafin, but not the determination of physiology of elafin. (**B**) HFD-treated mice were treated with either oral gavage of Elafin-Eudragit formulation (10 mg/kg) daily or subcutaneous injection of PEG-elafin (3.25 mg/kg) every 48 h for 14 days. Serum elafin levels. (**C**) Serum leptin levels. (**D**) Serum IFNγ and IL-1β levels. (**E**) Daily food consumption. (**F**) Change in fat mass. (**G**) Change in body weight over 14 days. (**H**) Fasting blood glucose levels. Each group consists of 8 mice.

The subcutaneous injection of natural elafin to RD-treated male mice showed a short half-life (3 h) in circulation (Fig. 6A). We generated PEGylated elafin for subcutaneous injection and elafin-Eudragit formulation for oral administration. Both subcutaneous and oral administration of the modified elafin formulations produced comparable serum elafin levels as lentiviral elafin overexpression in the HFD-treated male mice (Fig. 6B). Both elafin delivery approaches also significantly increased circulating leptin levels (Fig. 6C) and significantly reduced circulating IFNγ levels, food consumption, fat mass gain, fasting blood glucose levels, and liver steatosis of the HFD-treated male mice (Fig. 6D–H and S6F). Also, oral elafin-Eudragit administration, but not subcutaneous PEG-Elafin injection, reduced body weight gain in the HFD-treated male mice (Fig. 6G).

## Discussion

This report is the first to demonstrate the reduced circulating elafin levels in men with T2DM (Fig. 1A). The reason behind the gender difference in serum elafin levels in non-diabetic and prediabetic patients is unknown. Interestingly, a previous study demonstrated that female sex hormone (estradiol) suppressed elafin secretion in human vaginal epithelial cells^41^. Another study showed that estrogen receptor-positive breast cancer tumors have lower elafin mRNA expression than estrogen receptor-negative counterparts^42^. Estrogen, via estrogen receptor, inhibits elafin expression in human cells that may lead to reduced circulating elafin levels in women without prediabetes/diabetes and women with prediabetes. On the other hand, the low serum elafin levels in patients with T2DM are gender independent (Fig. 1A). We speculate that the inhibitory effect of estrogen becomes trivial when the serum levels in women with diabetes are already low.

Serum elafin levels are not correlated with BMI in men with T2DM (Figure S1B), possibly because BMI is not an ideal indicator for obesity among patients with abnormal blood glucose levels^43^. In this study, fat mass and calorie intake were not determined among patients with T2DM. Interestingly, elafin overexpression and elafin-LV exosome injection caused a statistically significant reduction of fat mass and an insignificant reduction in body weight of mice (Figs. 2B,C, 5B,C). The change of fat mass (less than 3.5% range) had a minor impact on the body weight because HFD-treated male mice have 20% fat mass and 80% lean mass, while RD-treated male mice have 10% fat mass and 90% lean mass.

This study is the first to illustrate the elafin-dependent regulation of food consumption via the immune system in mice (Fig. 3). We speculate that the decreased circulating elafin levels in men with T2DM are caused by reduced elafin expression in immune cells, as exposure to serum exosomes from patients with T2DM inhibited elafin mRNA expression in PBMCs (Figure S4F). PBMCs consist of a significant portion of T and B lymphocytes. Exposure to serum exosomes from patients with T2DM and high serum elafin levels significantly increased miR181b-5p and miR210-3p expression in PBMCs (Figure S4G). This finding indicates that circulating immune cells produce these elafin-regulated miRNAs and supports the positive correlation between serum elafin levels and serum exosomal miR181b-5p and miR210-3p in men with T2DM (Fig. 4C).

Elafin overexpression led to a similar pattern of increased miR181b-5p, miR210-3p, and miR219-5p expression in both circulating immune cells and serum exosomes in the HFD-treated mice (Fig. 4A,B), while transplantation of these elafin-conditioned splenocytes and serum exosomes were equally effective in reducing food intake in HFD-treated male recipient mice (Figs. 3, 5). Both mouse studies suggest that these appetite-regulating serum exosomal miRNAs are derived from splenocyte-derived immune cells. Lymphocytes are capable of secreting exosomal miRNAs that modulate diabetes development^44^. The elafin-dependent regulation of food consumption in the HFD-treated mice should involve T- and B-lymphocytes because they constitute a majority of splenocytes and have a long lifespan (several weeks). Splenic dendritic cells are a relative minority of splenocytes and last only a few days.

The disruption of elafin-dependent anti-obesity, anti-diabetic, and hepatoprotective effects in leptin-deficient *ob/ob* male mice reflect their absolute dependency on leptin (Fig. 2B,E and S5D). Humans and mice have differences in gene expression and physiological responses. For example, elafin-dependent miR181b-5p and miR210-3p are associated with leptin in men with T2DM (Fig. 4E), while elafin regulates leptin expression via miR181b-5p and miR219-5p in HFD-treated male mice (Fig. 5). The correlation between serum elafin, cytokines (IFNγ), and exosomal miRNAs (miR181b-5p) in men with T2DM are similarly reflected by the elafin-mediated inhibition of IFNγ expression and promotion of miR181b-5p expression in HFD-treated male mice (Figs. 3, 4 and S4A–E).

Elafin overexpression inhibited hyperphagia and promoted leptin-mediated suppression of food consumption in the HFD-treated male mice, which were reversed by IFN-γ injection (Figs. 3F, 5J). IFNγ deficiency is associated with the improvement of leptin sensitivity. For example, RD-treated IFNγ deficient male mice have reduced food consumption^45^^,^ while HFD-treated IFNγ deficient mice have improved insulin sensitivity, reduced adipocyte diameter, and lowered serum leptin levels^46,47^. These findings suggest that elafin improves leptin sensitivity by inhibiting IFNγ expression in HFD-treated male mice.

Although miR181b-5p and miR219-5p are known to regulate glucose homeostasis and obesity respectively^48,49^, our study is the first to address how elafin-dependent miR181b-5p and miR219-5p regulate adipose leptin expression (Fig. 4G). Consistent with our finding (Figure S4E), miR181b also inhibits IFNγ expression in human CD4 Th1 lymphocytes^50^. As HFD-treated mice are leptin resistant^51^^,^ elafin-dependent immune-derived serum exosomal miR181b reduces IFNγ mRNA expression, lowers circulating IFNγ levels, and subsequently improves leptin sensitivity (Figs. 3A,F, 4A, and S6D). At the same time, elafin-dependent miR181b-5p and miR219-5p induce leptin expression in human and mouse adipocytes (Fig. 4G and S3D). These two co-existing complementary pathways enable elafin to restore the leptin pathway and suppress food consumption.

Although elafin inhibits IL-1β expression in the circulating immune cells of HFD-treated mice (Fig. 3A), elafin does not improve leptin sensitivity via IL-1β inhibition because IL-1β injection did not increase food consumption in the elafin-overexpressing mice (Fig. 3F). The exacerbated hyperglycemia in the IL-1β-treated elafin-overexpressing mice may reflect IL-1β-induced insulin resistance in adipocytes (Fig. 3F)^52^.

The elafin-dependent mesenteric fat and hepatic CD36 expression are associated with obesity and liver steatosis in mice (Figure S2E, right panel, S3A, and S6A-C). However, elafin, miR181b-5p mimic, or miR219-5p mimic did not directly alter Cd36 mRNA expression in the adipocytes in vitro (Fig. 4G). We speculate that elafin-dependent CD36 expression is regulated by food consumption of the mice as adipose and hepatic Cd36 mRNA expression varies upon calorie intake^53,54^.

As liver steatosis in HFD-treated mice is CD36-dependent^38^^,^ the elafin-driven reduction of hepatic Cd36 mRNA expression should mediate the inhibition of liver steatosis (Figure S6A). Elafin may not affect cholesterol synthesis in the liver because elafin overexpression did not affect hepatic HMG-CoA reductase mRNA expression in HFD- and HCD-treated mice (Figure S6G). HFD-treatment activated a mixed population of resident Kupffer cells and recruited macrophages^55^^,^ as reflected by significantly increased hepatic F4/80 mRNA expression in the HFD-treated mice (Figure S6H).

Hepatic IL-6 expression is positively correlated with the severity of nonalcoholic steatohepatitis (NASH) in patients^56^^,^ while hepatic TNF expression is associated with liver fibrosis among patients with NASH^57^. Consistent with our microscopic observations (Figure S5–S6), the significantly increased hepatic Il6, but not TNF, mRNA expression indicated the presence of hepatic inflammation without fibrosis in the HFD-treated mice (Figure S6I). The exogenous elafin-regulated inhibition of hepatic F4/80 mRNA expression, along with the modestly reduced hepatic pro-inflammatory cytokine (Il6) mRNA expression, might reflect the amelioration of hepatic inflammation (Figure S6I).

Liver enzyme levels may reflect liver injury among patients. There is no correlation between serum elafin levels and liver enzyme levels (ALT, AST, and ALP) among patients with T2DM (Figure S7A–F). The presence of non-alcoholic fatty liver disease (NAFLD) is independent of serum elafin levels, severity of diabetes, or liver enzyme levels among patients with T2DM (Figure S7G).

Adipose tissue depots in obese mice consist of more than 50% F4/80+ macrophages^58^^,^ which are associated with obesity and insulin resistance^59^. A previous mouse study also showed that macrophages, instead of adipocytes, express almost all of the adipose tissue-derived TNFα and a significant part of adipose tissue-derived IL-6^58^. However, elafin overexpression did not affect F4/80, Tnf, and Il-6 mRNA expression in mesenteric, epididymal, and subcutaneous fat of HFD-treated mice (Figure S3A). Furthermore, elafin did not affect lipopolysaccharide (LPS)- and palmitate-induced TNFα secretion in mouse RAW264.7 macrophages (Figure S3E). These findings suggest that elafin is unlikely to affect adipose tissue macrophage accumulation and activation in HFD-treated mice.

Given the large proportion of macrophages in the adipose tissues of obese mice^58^^,^ elafin overexpression did not affect miR181b-5p, miR210-3p, and miR219-5p expression in mesenteric and epididymal fat tissues of HFD-treated mice (Figure S3C). This evidence suggests that elafin does not regulate the expression of these miRNAs in the adipose macrophages and adipocytes of HFD-treated mice.

Consistent with the increased leptin mRNA expression in the mesenteric fat of HFD-treated mice (Figure S2E), leptin mRNA expression in mesenteric fat is positively correlated with the BMI values of patients without diabetes (Figure S8A–B). Adipose elafin mRNA expression and miR181b-5p, miR210-3p, and miR219-5p expression are independent of the BMI values of these patients (Figure S8C). Adipose miR181b-5p, miR210-3p, and miR219-5p expression and leptin mRNA expression are not associated with adipose elafin mRNA expression in patients without diabetes, as shown by low R^2^ values (Figure S8D–E).

Although systemic injection of elafin (200 mg per subject) was well tolerated in humans^60^, formulation optimization is necessary to overcome the short half-life of elafin in circulation (Fig. 6A). For example, PEGylation extends the half-life of subcutaneously injected drugs in circulation. Alternatively, Eudragit coating protects drugs from gastric acid inactivation and releases it in the alkaline terminal ileum and colon, which is common in orally-active medications for gastrointestinal and metabolic diseases^61,62^. As both subcutaneous PEG-elafin and oral Elafin-Eudragit formulations mimicked the protective effects of elafin overexpression (Fig. 6), they should be clinically useful for reversing diabetes.

In conclusion, circulating elafin levels are reduced and inversely correlated with hyperglycemia in men with T2DM. As shown by our mouse studies, elafin inhibits hyperglycemia via pleiotropic mechanisms. Elafin overexpression increases leptin sensitivity by suppressing immune cell-derived IFNγ in HFD-treated male mice. Elafin overexpression also induces appetite-lowering leptin expression in mesenteric fat via immune cell-derived exosomal miR181b-5p and miR219-5p expression in the HFD-treated male mice. Leptin-mediated reduced food intake subsequently inhibits obesity, hyperglycemia, and liver steatosis in HFD-treated male mice. Subcutaneous PEG-Elafin injection and oral Elafin-Eudragit formulation administration are also effective against obesity, hyperglycemia, and liver steatosis in HFD-treated male mice. The discoveries of this study may prove to be vital in improving the management of diabetes.

## Methods

### Human serum samples

Patients were assigned to non-diabetic, prediabetes, and diabetic groups using the American Diabetes Association criteria^63^. UCLA pathology laboratory collected blood from patients and prepared the sera by centrifugation, following its standard operating procedures (SOPs). Inclusion criteria: Subjects 20–70 years of age. Exclusion criteria: Pregnant women, prisoners, or minors under 18 years old were excluded. Patients with type 1 diabetic mellitus (T1DM) were excluded. This study was a prospective study. The baseline characteristics are shown in Table S1.

The mean values of blood tests including creatinine (Cr), blood urea nitrogen (BUN), alanine aminotransferase (ALT), aspartate aminotransferase (AST), alkaline phosphatase (ALP), and albumin (ALB) are within normal ranges (Table S1). A woman without diabetes, a man with prediabetes, three men with T2DM, and a woman with T2DM were diagnosed with non-alcoholic fatty liver disease (NAFLD) (Table S1). No NASH, hepatic fibrosis, or other hepatic abnormalities are noted in the clinical data among all other patients.

All patients with T2DM are not treatment naïve. 82%, 18%, and 5% of the patients with T2DM used metformin, sulfonylureas, and insulin, respectively. None of the non-diabetic or prediabetic patients used metformin, sulfonylureas, or insulin. There is no strong correlation between the use of anti-diabetic drugs and serum elafin levels and the gender of the patients with T2DM (Table S2).

### ELISA measurement of human serum samples

Elafin (DY1747, R&D Systems), adiponectin (DY1065, R&D Systems), insulin (#90095, Crystal Chem), and leptin (#80968, Crystal Chem) levels were determined using ELISA. Human serum cytokine levels were determined with a 27-plex Multiplex ELISA (#m500kcaf0y, Bio-Rad) using a Bio-plex 3D suspension array system (Bio-Rad) at UCLA Center for Systems Biomedicine^64^.

### Human mesenteric fat samples

Human mesenteric fat tissues from non-diabetic patients were collected from the Cedars-Sinai Medical Center (CSMC) between 2010–2014 prospectively. Inclusion criteria: The CSMC’s gastroenterologists referred the patients to surgical procedures, as medically indicated. Patients with colorectal cancer, inflammatory bowel diseases, intestinal perforation, diverticulitis, colonic inertia, or obesity were included. Exclusion criteria: Pregnant women, prisoners, or minors under age 18 were excluded. Patients with concurrent acute infection (CMV, *C. difficile,* and tuberculosis) were excluded. Patients with prediabetes or diabetes (T1DM or T2DM) were not included. The baseline characteristics are shown in Table S3.

### Animal experiments

Eight-week-old male C57BL/6J mice (stock #000664), *Rag*^−/−^ mice (stock #002216), and *ob/ob* mice (stock #000632) were purchased from Jackson Laboratories and maintained at the UCLA animal facility under standard environmental conditions with a 12-h light period and a 12-h dark period per day at 25 °C room temperature. They were housed in disposable plastic cages with HEPA filtered air circulation, autoclaved bedding, animal chow, and sterile water ad libitum^5^*.* Mice were fed with either regular diet (RD) (6% fat, #7013, Harlan Laboratories), high-fat diet (HFD) (45% Kcal from fat; #D12451, Research Diets, Inc.), or high-cholesterol diet (HCD) (Clinton/Cybulsky low-fat diet with 2% cholesterol, #D01101902C, Research Diets, Inc.) ad libitum. The leptin-deficient *ob/ob* mice were fed with regular chow ad libitum. All mice were randomized and allocated to each cage (4 mice per cage) by animal facility personnel before experiments began.

For systemic elafin overexpression, some groups were injected with control lentivirus (PS100064V, Origene) or elafin-overexpressing lentivirus (RC203136L1V, Origene) intravenously via tail veins (10^7^ infectious units per mouse). Some groups were injected with IL-1β protein 2.5 µg per mouse (#211-11B Peprotech) or IFNγ protein 5 µg per mouse (#315–05 Peprotech) intraperitoneally to increase circulating IL-1β or IFNγ levels.

Leptin sensitivity tests were performed by injecting 1 µg/kg leptin intraperitoneally (#450-31, Peprotech) daily for 3 days. At the endpoint of the experiments, mice were fasted for 6 h and tested for fasting blood glucose and cholesterol levels with a drop of tail vein blood using Freestyle Lite blood glucose meter and test strips (Abbott Diabetes Care) and Accutrend Plus meter and test strips (Roche), respectively. The oral glucose tolerance test (OGTT) was performed by feeding D-glucose (1 g/kg) to fasted mice via oral gavage, and the blood glucose level was measured 2 h after oral glucose feeding. Blood glucose and cholesterol levels are presented as mg/dL. Measurements of fasting blood glucose levels, blood total cholesterol levels, food consumption, and fat/lean mass (EchoMRI) were performed as described in our previous report^5^.

Free fatty acid levels in blood were determined by a free fatty acid quantification kit (#ab65341, Abcam). Mouse serum insulin (#90080, Crystal Chem), adiponectin (MRP300, R&D Systems), and leptin (#90030, Crystal Chem) levels were determined by ELISAs. Mouse serum cytokine levels were determined with a 23-plex Multiplex ELISA (#M60009RDPD, Bio-Rad) using a Bio-plex 3D suspension array system (Bio-Rad) at UCLA Center for Systems Biomedicine^64^.

### Splenocyte transplantation in mice

The 8-week-old HFD-treated male C57BL/6J mice infected with either control lentivirus or elafin-expressing lentivirus were used as donor mice. Spleens of donor mice were gently minced with a syringe pistol in 2 mL ice-cold phosphate-buffered saline (PBS). The splenocytes were filtered through 70 µm cell strainer with 10 ml ice-cold PBS. The cells were centrifuged (1,500 rpm) and resuspended in 200 µL ice-cold PBS. The splenocytes were then injected into HFD-treated male *Rag*^−/−^ recipient mice intraperitoneally^65^. The splenocytes from one donor mouse were injected into one recipient mouse.

### Cecal microbiota transplantation in mice

The 8-week-old HFD-treated male C57BL/6J mice with infection of either control lentivirus or elafin-expressing lentivirus were used as donor mice. Cecal content from donor mice was collected during dissection and immediately homogenized in ice-cold PBS (1 g/mL), followed by low-speed centrifugation for one minute. The supernatant of cecal material from one donor mouse was transferred to an 8-week-old HFD-treated male C57BL/6J recipient mouse via oral gavage daily (100 µL/mouse/day) for 14 days^61^.

### Mouse blood cell and serum exosome preparation and transplantation

Blood samples were collected in tubes containing 50 µL/tube of 0.5% ethylenediaminetetraacetic acid (EDTA) at the time of donor mouse dissection. The blood samples were centrifuged at 10,000 g for 5 min at 4 °C. The supernatant (serum) samples were used for ELISA assays and serum exosomes extraction. The pellets (blood cells) were resuspended in 10 mL 1X red blood cell lysis buffer (#420301, BioLegend) for 10 min, followed by dilution in PBS. The cell suspension was centrifuged at 8,000 rpm for 5 min at 4 °C, and the supernatant was discarded. The immune cell-containing pellets were resuspended and lysed in Qiazol reagent (#79306, Qiagen) for RNA extraction and RT-PCR experiments.

Serum exosomes of donor mice were prepared using total exosome isolation reagent (#4478360, ThermoFisher), and their quantities were then determined by BCA protein assay. The serum exosomes were diluted in PBS and injected into recipient mice intravenously via tail veins (10 µg per mouse).

For inhibition of miRNAs, control (YI00199006), miR-181b-5p (YCI0201288-FZA), and miR-219-5p (YCI0201241-FZA) inhibitors (Qiagen) were dissolved in PBS. The miRNA inhibitors (10 mg/kg in 100 µL/mouse) were injected into the exosome recipient mice subcutaneously under brief isoflurane anesthesia, as recommended by Qiagen.

### Histological assessment of hepatic steatosis in mouse liver

Hepatic steatosis was assessed with a NAFLD score: parenchymal involvement by steatosis with < 5% = 0; 5–33% = 1; 33–66% = 2; and > 66% = 3^66^. Two locations per mouse were observed by two investigators in a blinded manner.

### Oral and subcutaneous administration of modified elafin to mice

Eight-week-old male c57BL/6J (JAX #000664) mice were fed with HFD for 8 weeks to induce obesity and hyperglycemia, followed by administration of Elafin-Eudragit formulation (10 mg/kg) via oral gavage daily or subcutaneous injection of polyethylene glycol conjugated (PEGylated) elafin (3.25 mg/kg) every 48 h to mice for 14 days. Oral Elafin-Eudragit formulation was made by Dr. Xingguo Cheng at the Southwest Research Institute (SWRI), Texas. Elafin was coated with Eudragit FS30D polymer. This pH-responsive polymer is insoluble in acid but dissolves in a mildly alkaline environment (i.e., pH 7 or above), which is optimal for colonic delivery. Elafin-Eudragit was packaged into microparticles using an SWRI-patented spinning disk atomization technology. This packaging prevented leakage of elafin in acidic, aqueous solution. The oral elafin-Eudragit formulation was dissolved in mildly acidified (pH 5) water containing 0.5% hydroxypropyl methylcellulose (HPMC). PEGylated elafin was made by conjugation to methoxyl-PEG12 (New England Peptide Company). Control groups received either oral Eudragit-HPMC solution or subcutaneous PEG injection.

### Cell culture experiments

Mouse 3T3-L1 preadipocytes (#CL-173, ATCC) were cultured in Dulbecco's modified Eagle's medium (DMEM) (#11965-084, ThermoFisher) with 10% fetal bovine serum (FBS) (#10437-028, Life Technologies) and 1% penicillin/streptomycin/glutamine (P/S/G) (#10378-016, Life Technologies) mixture. The preadipocyte differentiation method was described in our previous report^5^. Differentiated 3T3-L1 adipocytes were serum-starved overnight followed by treatment with 10 ng/ml elafin (E7280, Sigma), 10 µg/ml exosomes, or 75 ng/ml miRNA mimics to study the role of elafin and its dependent molecules in lipid accumulation and gene expression. Control mimic (#479904-001, Qiagen), miR181b-5p mimic (MSY0000673, Qiagen), miR210-3p mimic (MSY0000658, Qiagen), and miR219-5p mimic (MSY0000664, Qiagen) were transiently transfected to 3T3-L1 adipocytes via HiPerfect transfection reagent (#301704, Qiagen) (75 ng/ml via overnight transfection). Adipocyte lipid accumulation was determined using Oil Red O staining, as described previously^5^.

Mesenteric fat preadipocytes from colon cancer patients were collected from a previous study and stored in liquid nitrogen^67^. The human preadipocytes were thawed and cultured in DMEM/F12 media containing 10% calf serum and 1% P/S (Invitrogen) until > 60% confluence was achieved. The preadipocytes were passaged to 6-well plates (400,000 cells/plate) in DMEM/F12 media containing 10% calf serum and 1% P/S. Two days later, the preadipocytes underwent a differentiation process, as described by our previous report^5^. The differentiated adipocytes were serum-starved for 6 h, followed by transient transfection with control, miR181b-5p, or miR219-5p mimics (75 ng/ml) via Lipofectamine 3,000 (L3000001, ThermoFisher) overnight. The transfected cells were then incubated in serum-free DMEM media for 6 h. The conditioned media were collected for leptin ELISA.

Primary human peripheral blood mononuclear cells (PBMCs) were obtained from a healthy donor (C-12907, Promocell). The PBMCs in mononuclear cell medium (C-28030, Promocell) were incubated with 10 µg/ml of human serum exosomes in serum-free DMEM for 3–24 h. The human serum exosomes were obtained with 12 patients per disease group. At the end of the experiments, PBMCs were centrifuged, and the supernatant was removed. The PBMC pellets were resuspended and lysed in Qiazol reagent (#79306, Qiagen) for RNA extraction and RT-PCR experiments.

Mouse RAW264.7 macrophages (#TIB-71, ATCC) were cultured in Dulbecco's modified Eagle's medium (DMEM) (#11965-084, ThermoFisher) with 10% fetal bovine serum (FBS) (#10437-028, Life Technologies) and 1% penicillin/streptomycin/glutamine (P/S/G) (#10378-016, Life Technologies) mixture. The macrophages were serum-starved overnight, followed by exposure to fatty acid-free bovine serum albumin (BSA) (A7030, Sigma), sodium palmitate (P9767, Sigma), or lipopolysaccharide (LPS) (L5418, Sigma) for 2 h, and then the addition of elafin (10-1000 ng/ml) for additional 6 h. The cell-conditioned media were used for mouse TNFα ELISA (DY410, R&D Systems).

### Quantitative real-time reverse transcription-polymerase chain reaction (RT-PCR) and miRNA PCR array

Total RNA was isolated by RNeasy mini kit (#74104, Qiagen) and reverse transcribed into cDNA by a high-capacity cDNA RT kit (#4368814, ThermoFisher). Quantitative PCR reactions were run with Fast Universal PCR master mix (#4352042, ThermoFisher) in a Bio-Rad CFX384 using cataloged primers (ThermoFisher) for human elafin (Hs00160066_m1), human leptin (Hs00174877_m1), mouse *Cd36* (Mm00432403_m1), mouse adiponectin (Mm00456425_m1), mouse leptin (Mm00434759_m1), and mouse Tnf (Mm00443258_m1). Relative mRNA quantification was performed by comparing test groups and control group, after normalization with endogenous control gene human 18S (Hs99999901_s1) or mouse *Gapdh* (Mm99999915_g1)^68^.

For serum exosomal miRNA PCR array determination, serum exosomal total RNA was isolated with RNeasy mini kit (#74104, Qiagen) and reverse transcribed into cDNA by a miScript II RT kit (218161, Qiagen). PCR reactions were run with miScript SYBR Green PCR kit (218073, Qiagen) in a mouse-specific Serum and Plasma miScript miRNA PCR array (MIMM-106ZE-4, Qiagen), which detected 84 miRNAs. Relative miRNA quantification was performed by comparing test groups and control group, after normalization with housekeeping miRNAs.

For additional miRNA determination, RNA was reverse transcribed into cDNA by a miRCURY LNA RT Kit (339340, Qiagen). Quantitative PCR reactions were run with miRCURY LNA SYBR Green PCR kit (339345, Qiagen) in a Bio-rad CFX 384 using miRCURY PCR assays for miR-181b-5p (YP00204530), miR-210-3p (YP00204333), miR-219-5p (YP00204780), and RNU1A1 (YP00203909) from Qiagen Company.

The fold changes are expressed as 2^∆∆Ct^. Fold-change values greater than one indicate a positive- or an up-regulation, and the fold-regulation is equal to the fold-change. Fold-change values less than one indicate a negative or down-regulation, and the fold-regulation is the negative inverse of the fold-change.

### Power analysis

We performed power analyses once we began the experiments. For human serum experiments, we included at least six patients per group to achieve a statistically significant difference of circulating elafin between the non-diabetic group and the prediabetic group (9.6 vs. 10.8 ng/ml) with SD = 0.5, alpha = 0.05, and power = 0.8. For animal studies, we included eight mice per group to achieve a statistically significant difference in fat mass between HFD-treated mice and RD-treated mice (3.18% vs. 0.53%) with SD = 1.4%, alpha = 0.05, and power = 0.8. We did not perform power analysis for cell culture experiments but followed the common practice of performing in vitro experiments three times independently. ELISA and real-time RT-PCR experiments were performed in duplicate.

### Statistical analysis

Investigators, except Hon Wai Koon, were blinded to the group allocation. For cell culture experiments, we pooled data from multiple experiments. Bar graphs and scatter plots were made using Microsoft Excel. Equations and R^2^ values in scatter plots were generated by Microsoft Excel. Results were expressed as mean +/− SEM. Unpaired Student’s *t*-tests were used for two-group comparisons of continuous data (GraphPad QuickCalcs) online. One-way ANOVAs with Tukey Honestly Significant Difference *post-hoc* tests were used for multiple-group comparisons (Statpages) online. The odds ratio was calculated (Medcalc) online. Significant *p* values are shown in each figure.

### Ethics statement

All experimental protocols for human study were approved by Institutional Review Boards (IRBs) of the University of California Los Angeles (UCLA) and Cedar-Sinai Medical Center. Human serum samples were collected from the UCLA Ronald Reagan Medical Center and Division of Clinical Nutrition following procedures established by UCLA: IRB #13-001069 and IRB#14-000905 as described previously^5^. Separate informed consent was waived by UCLA IRB as the blood collection procedure was covered by a UCLA pathology’s written consent form. The mesenteric fat samples were collected from Cedars Sinai Medical Center following procedures established by Cedars-Sinai IRBs 3358 and 23705, and UCLA IRB 11-001527. Separate informed consent was waived by UCLA IRB as the fat sample collection procedure was covered by a Cedars-Sinai Medical Center’s written consent form. All methods in human subjects’ research were carried out in accordance with relevant guidelines (Declaration of Helsinki) and regulations. The UCLA Institutional Animal Care and Use Committee approved all procedures (#2007-116). All methods in animal research were carried out in accordance with relevant guidelines and regulations.

## Supplementary information

Supplementary Figure S1.

Supplementary Figure S2.

Supplementary Figure S3.

Supplementary Figure S4.

Supplementary Figure S5.

Supplementary Figure S6.

Supplementary Figure S7.

Supplementary Figure S8.

Supplementary Table S1.

Supplementary Table S2.

Supplementary Table S3.

Supplementary Legends.

## Data Availability

The data and materials used in this study are available at Dr. Koon’s laboratory.
