# Supplementary figures and images for: Elafin inhibits obesity, hyperglycemia, and liver steatosis in high-fat diet-treated male mice
Source: Sci Rep. 2020 Jul 30;10:12785. doi: 10.1038/s41598-020-69634-3 (PMC7393145; doi:10.1038/s41598-020-69634-3)

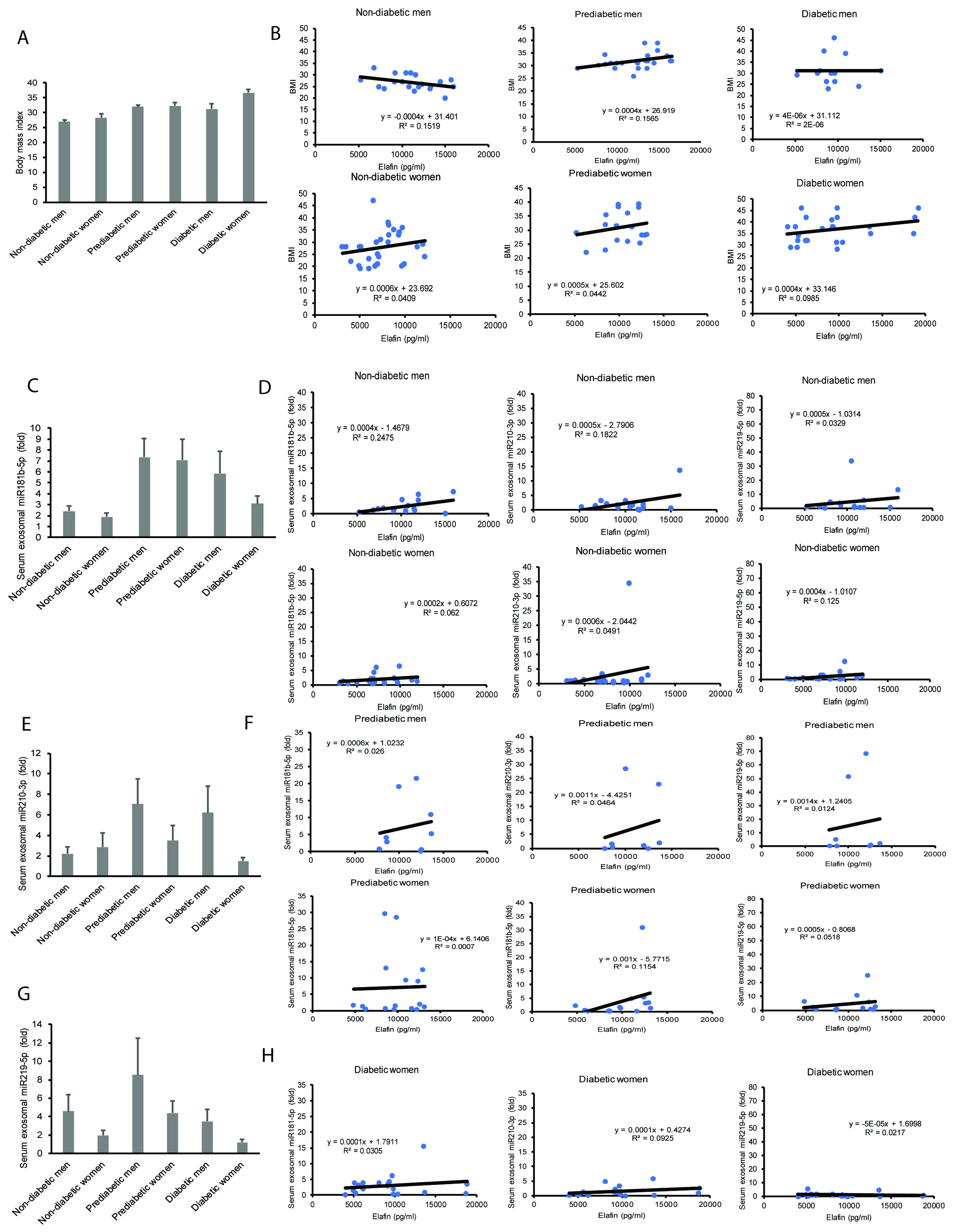

Supplement: Supplementary file 1 — Supplementary Figure S1. [file 41598_2020_69634_MOESM1_ESM.tif]

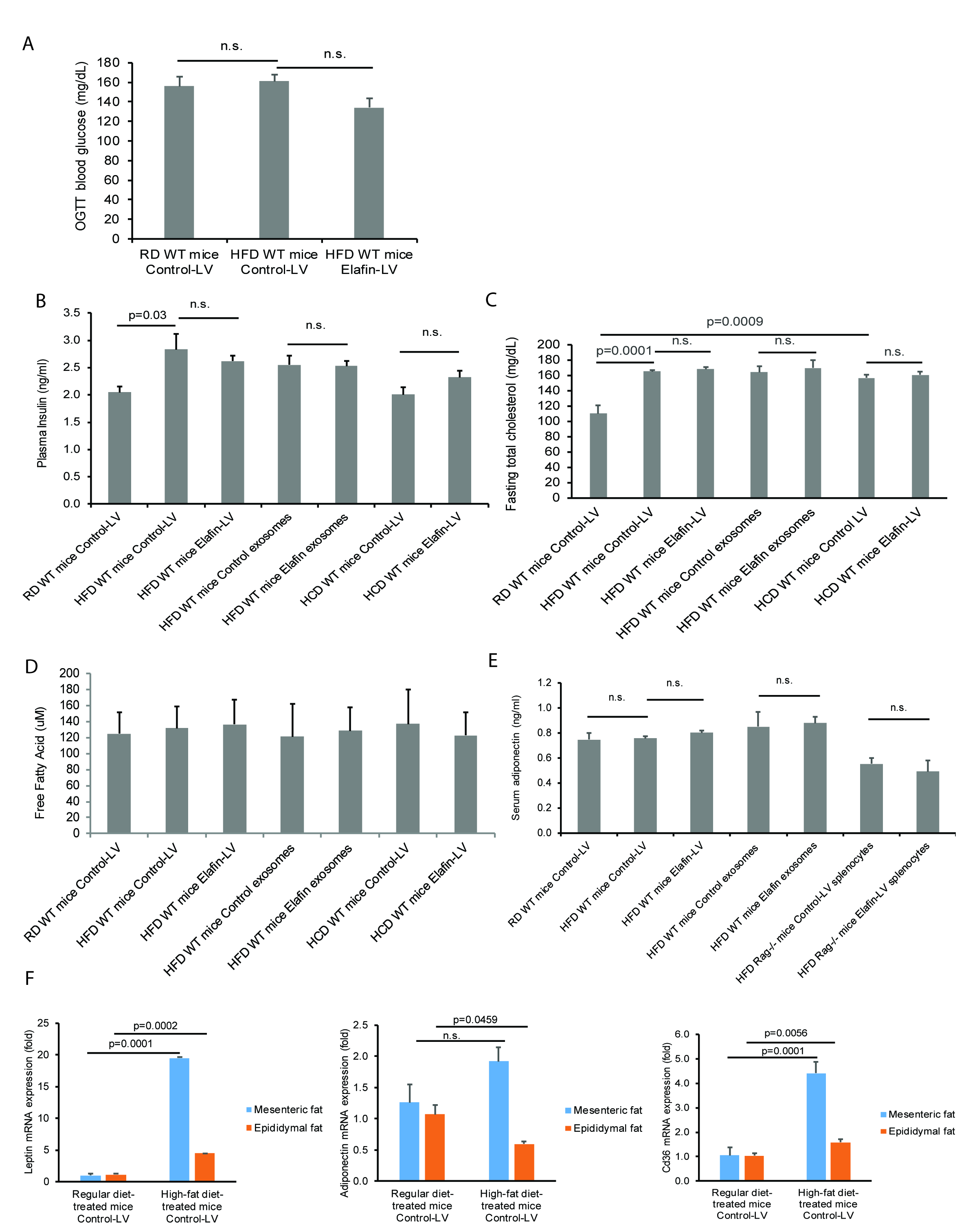

Supplement: Supplementary file 2 — Supplementary Figure S2. [file 41598_2020_69634_MOESM2_ESM.tif]

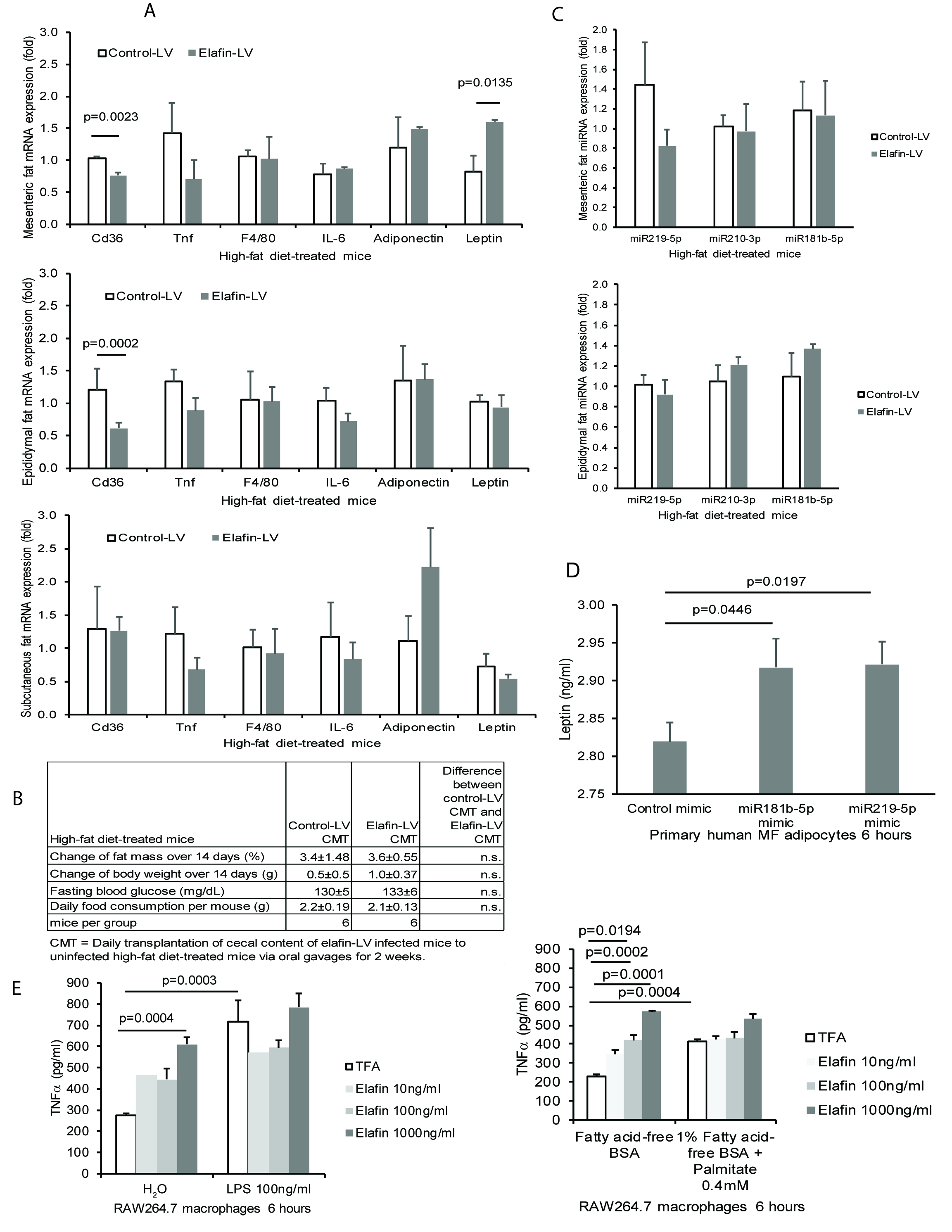

Supplement: Supplementary file 3 — Supplementary Figure S3. [file 41598_2020_69634_MOESM3_ESM.tif]

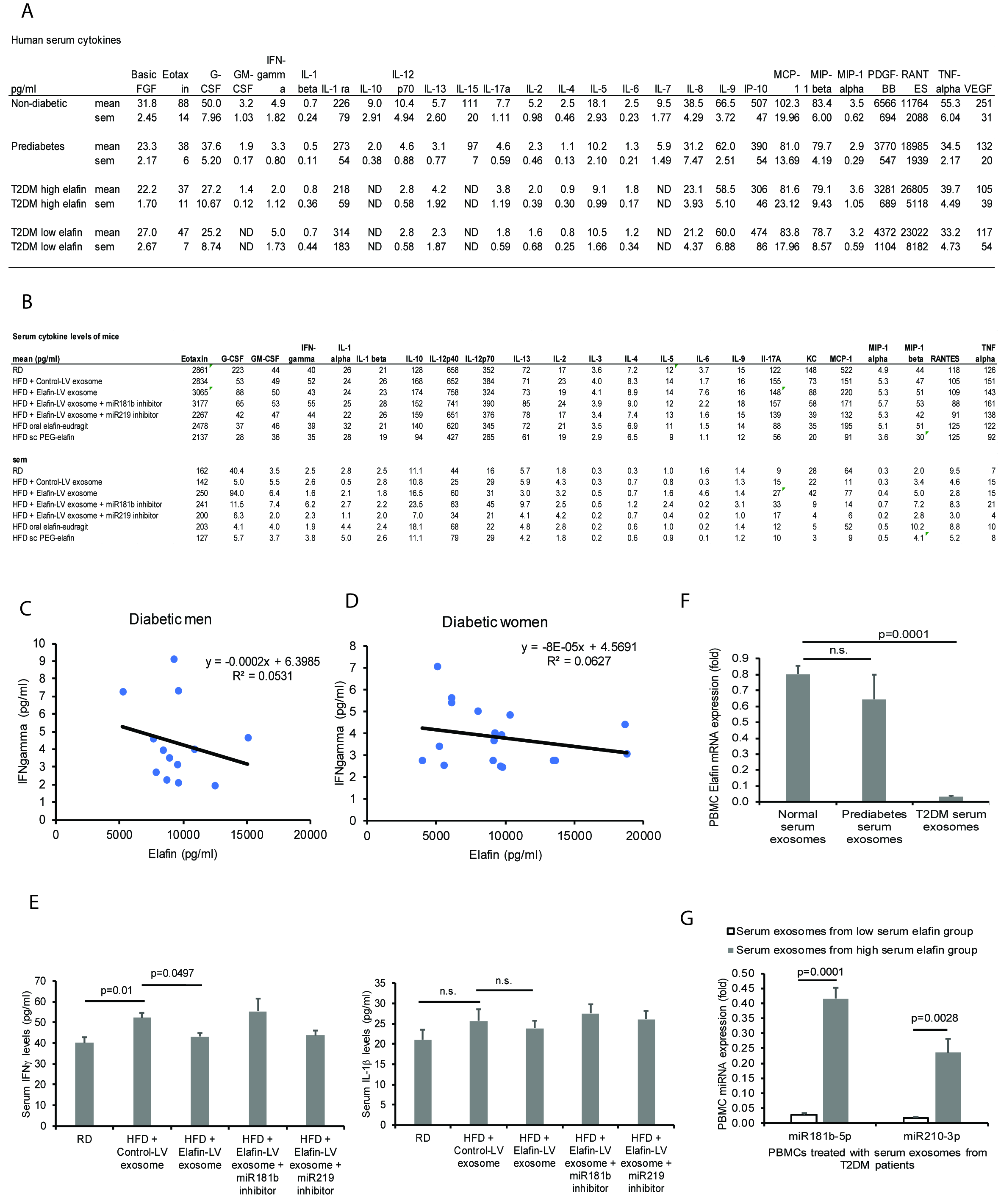

Supplement: Supplementary file 4 — Supplementary Figure S4. [file 41598_2020_69634_MOESM4_ESM.tif]

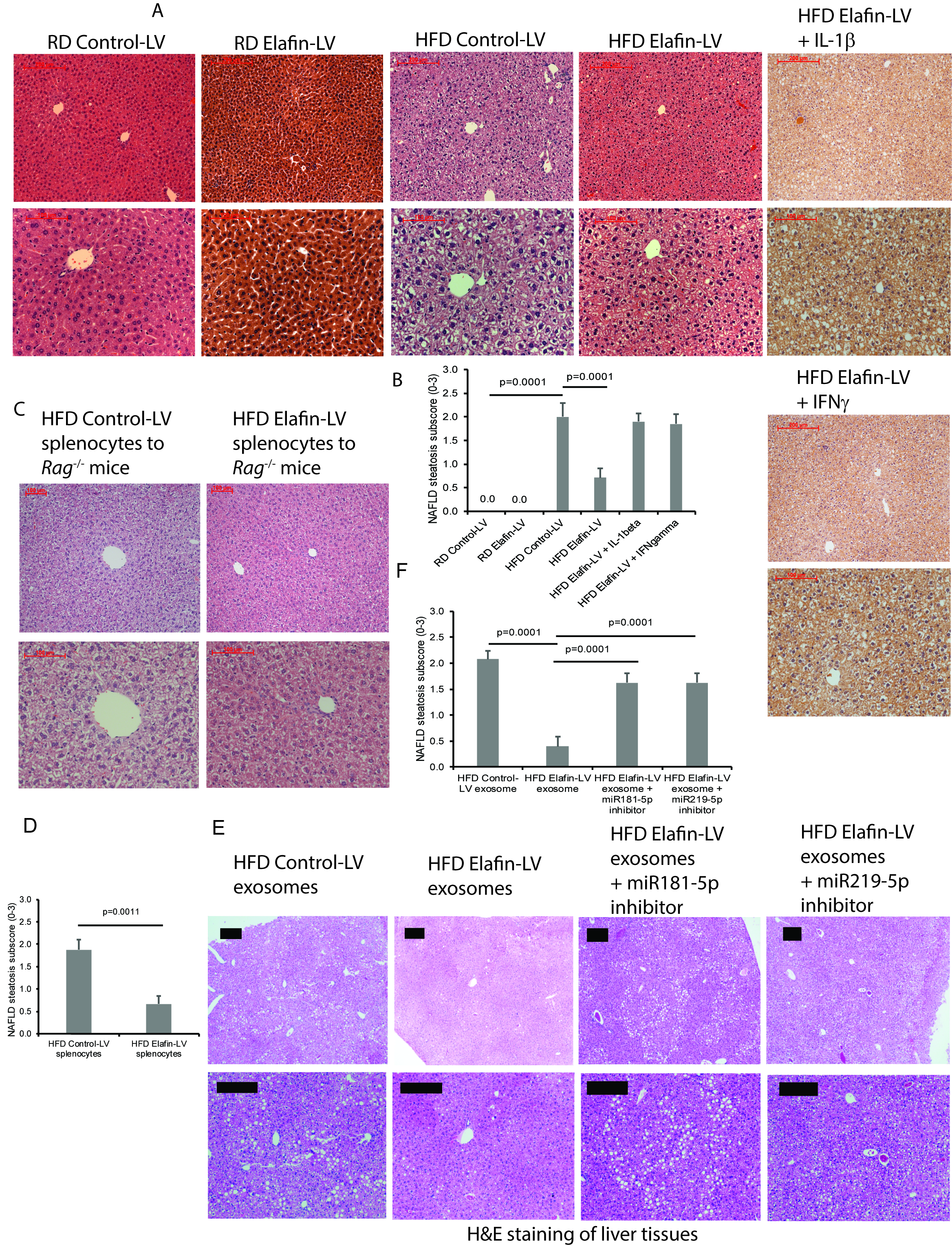

Supplement: Supplementary file 5 — Supplementary Figure S5. [file 41598_2020_69634_MOESM5_ESM.tif]

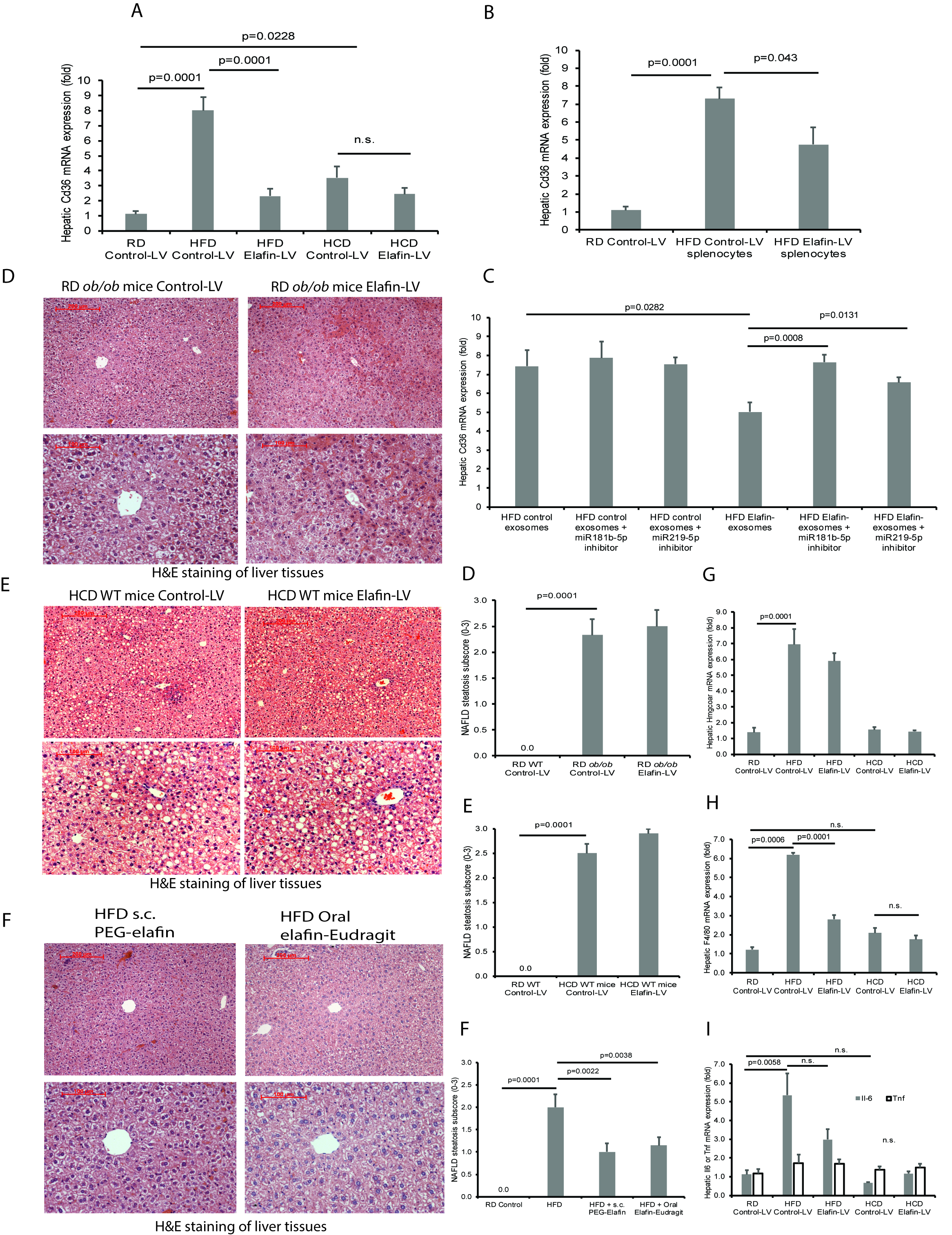

Supplement: Supplementary file 6 — Supplementary Figure S6. [file 41598_2020_69634_MOESM6_ESM.tif]

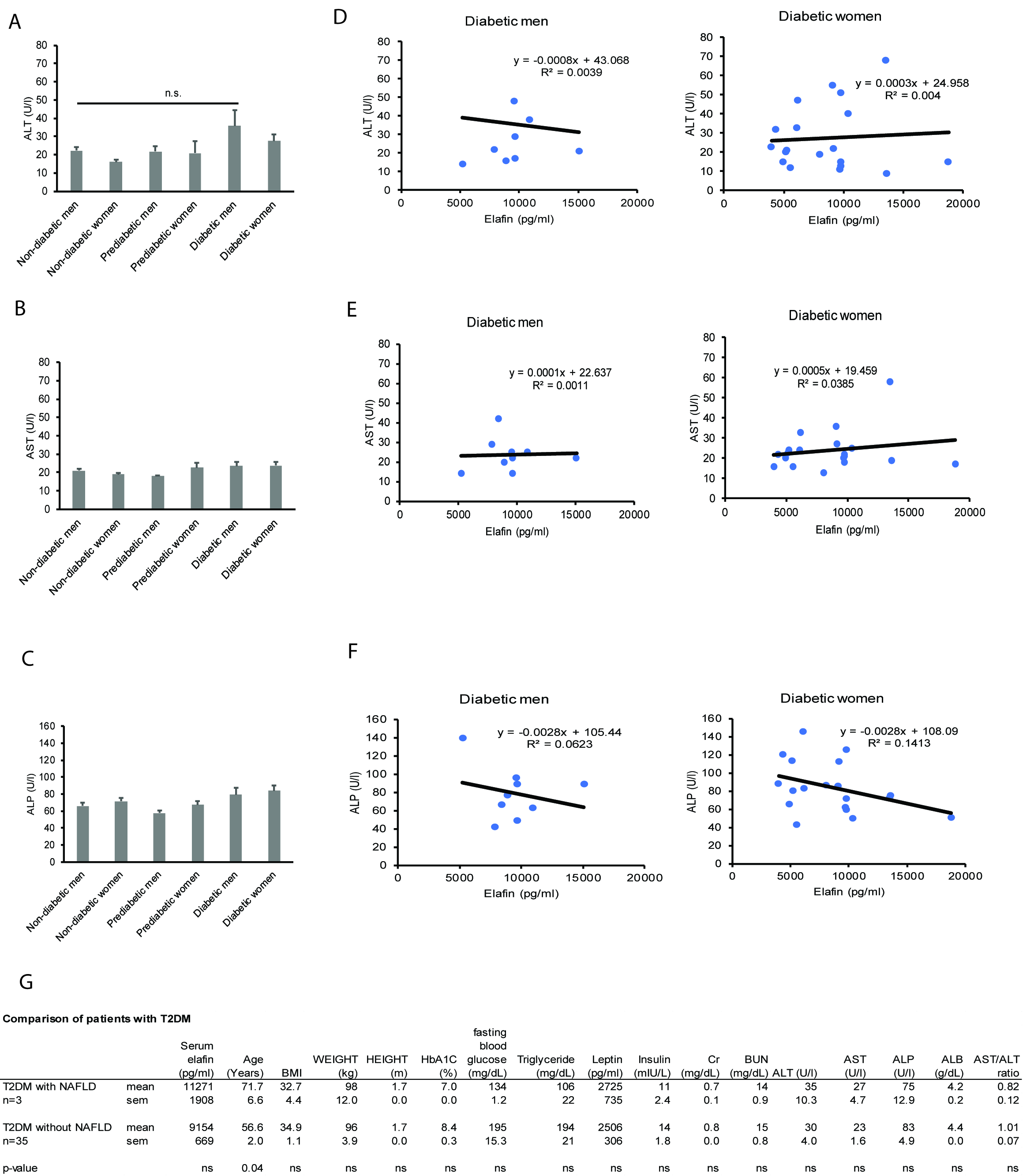

Supplement: Supplementary file 7 — Supplementary Figure S7. [file 41598_2020_69634_MOESM7_ESM.tif]

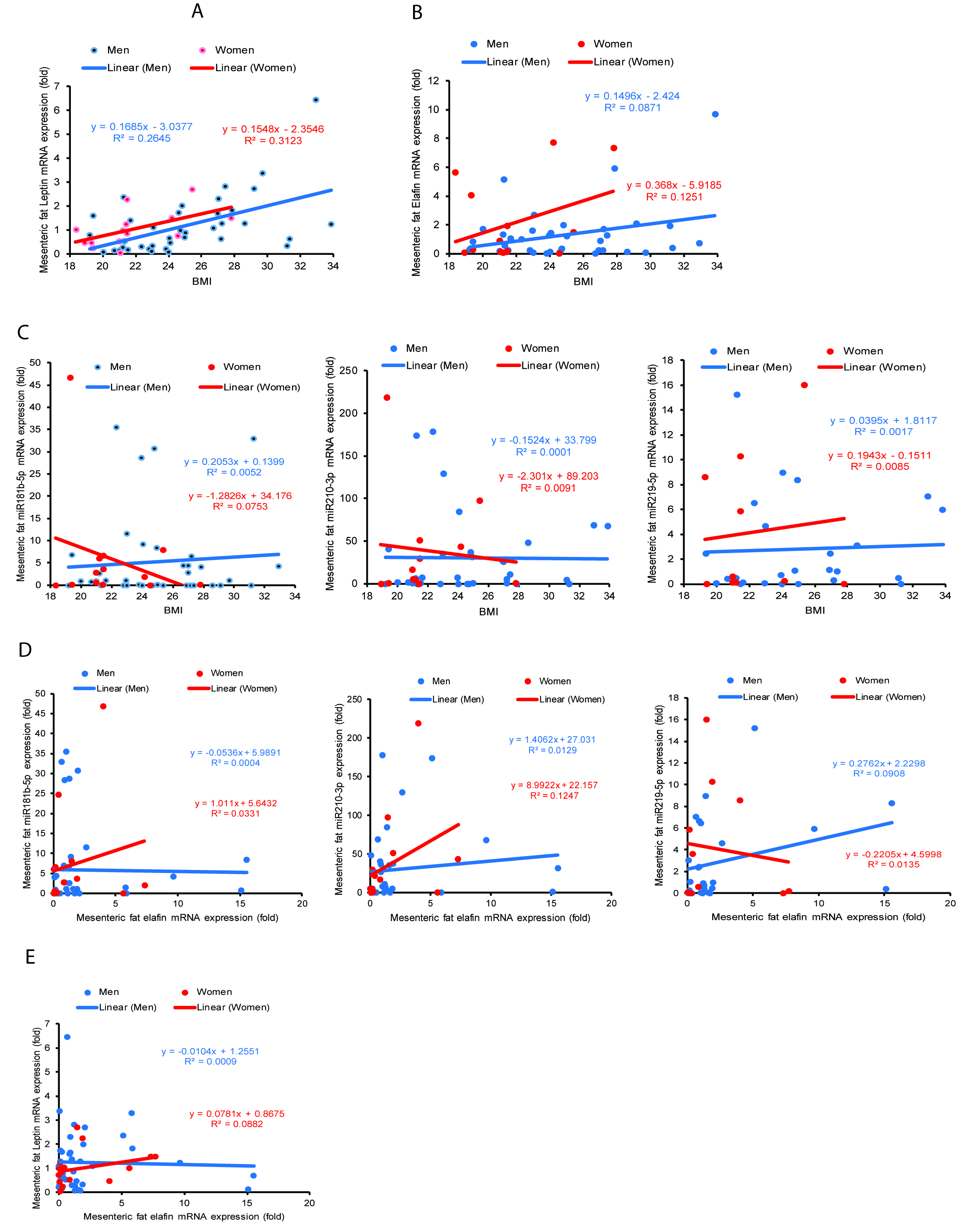

Supplement: Supplementary file 8 — Supplementary Figure S8. [file 41598_2020_69634_MOESM8_ESM.tif]

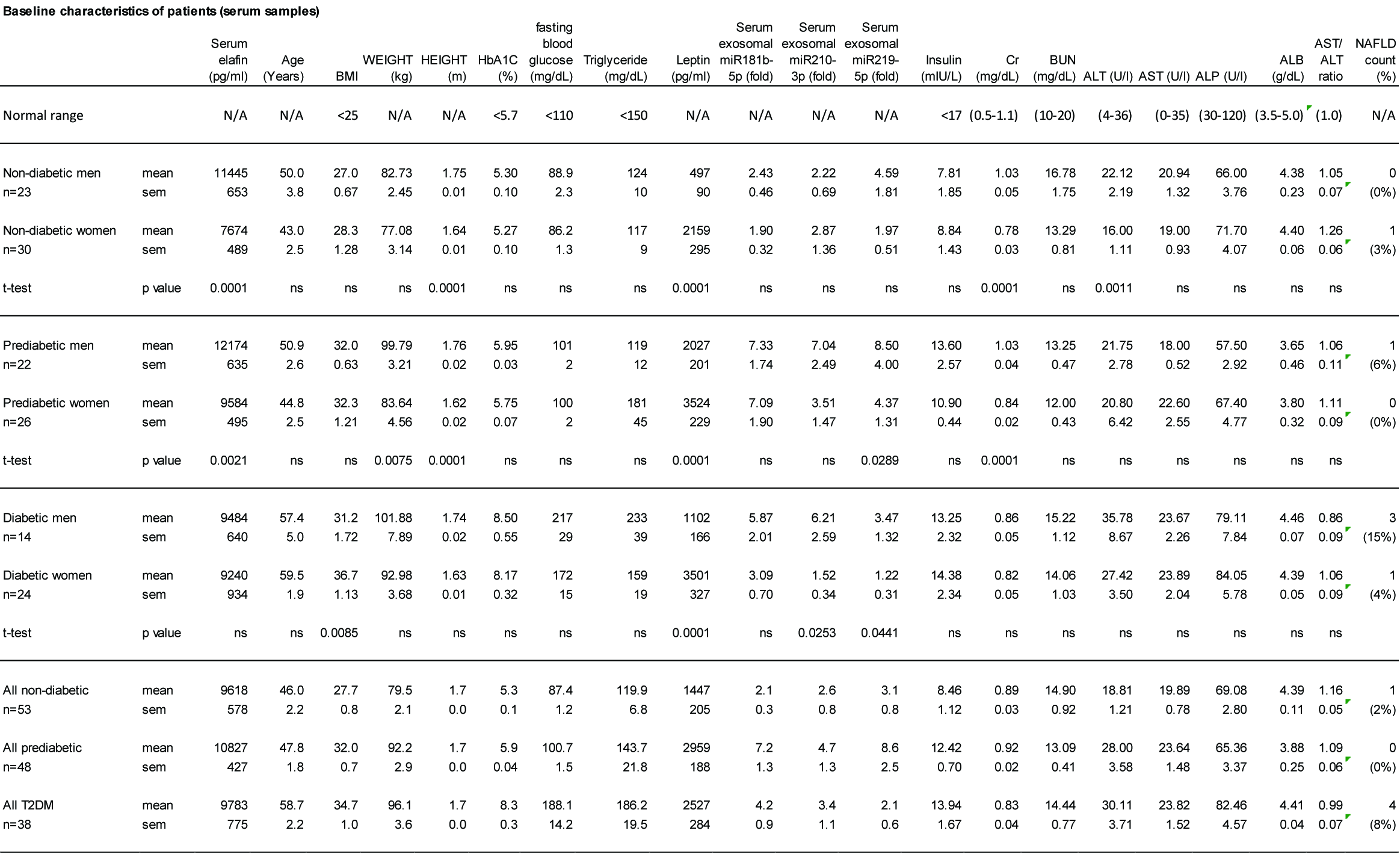

Supplement: Supplementary file 9 — Supplementary Table S1. [file 41598_2020_69634_MOESM9_ESM.tif]

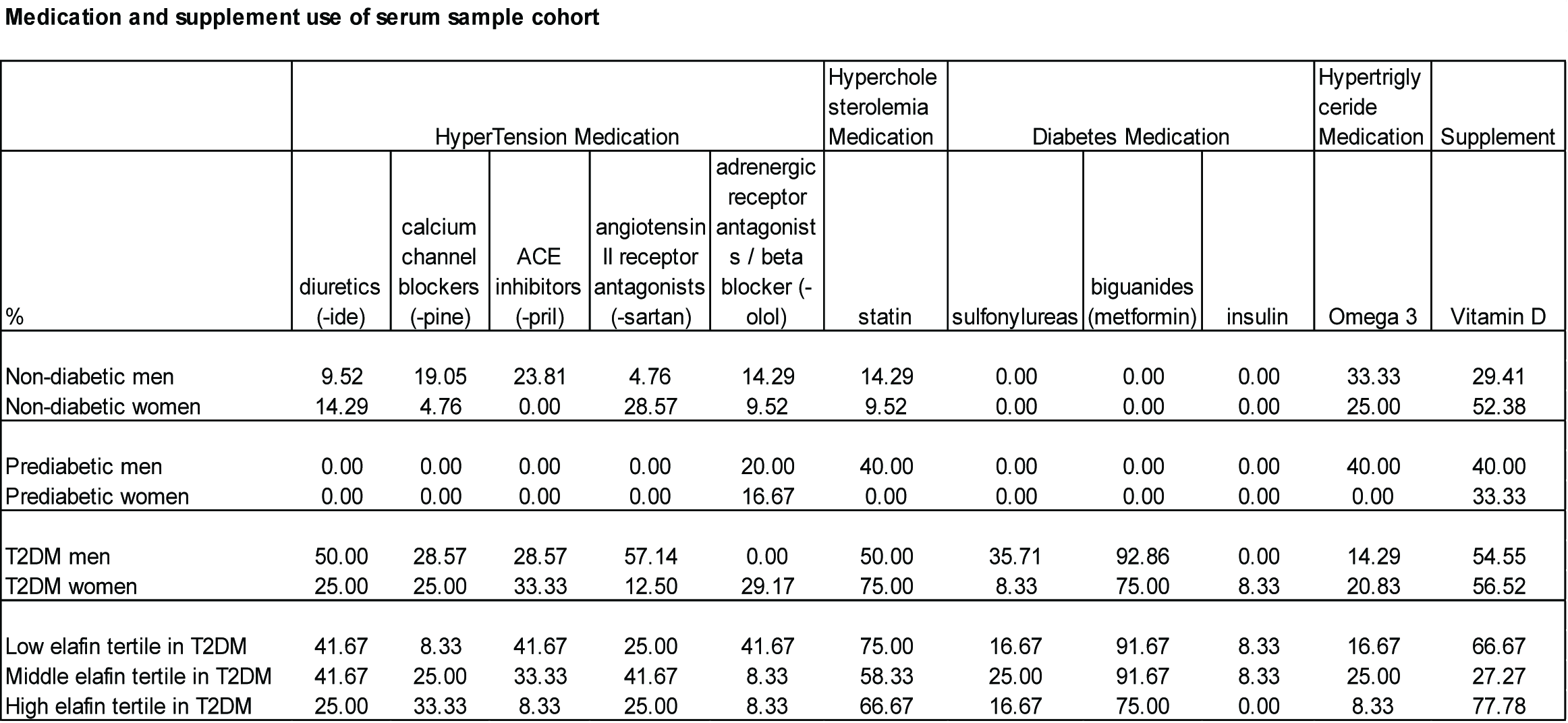

Supplement: Supplementary file 10 — Supplementary Table S2. [file 41598_2020_69634_MOESM10_ESM.tif]

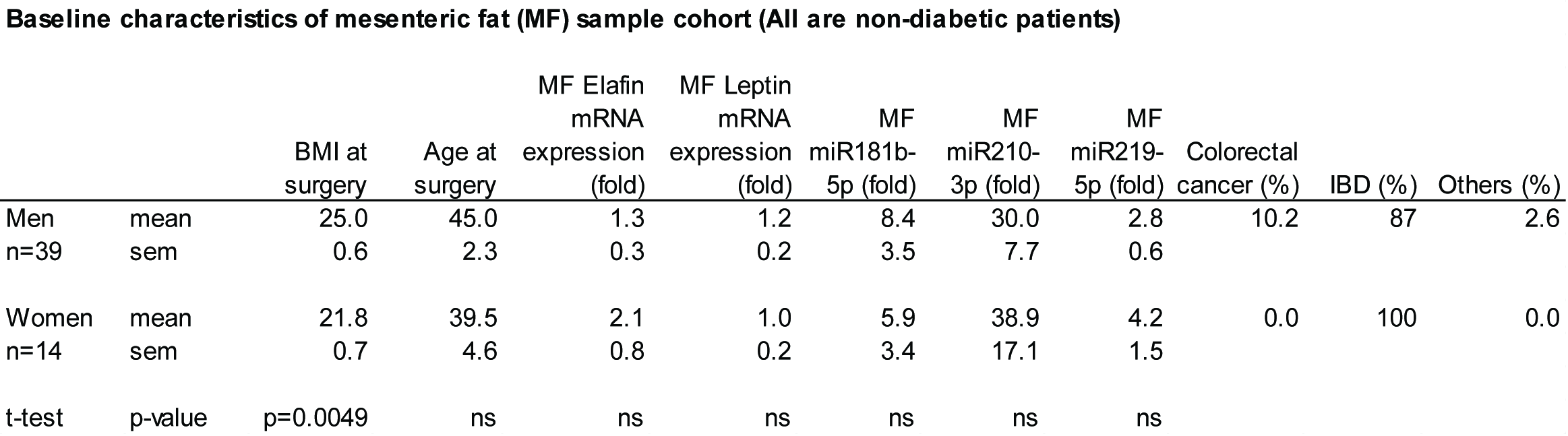

Supplement: Supplementary file 11 — Supplementary Table S3. [file 41598_2020_69634_MOESM11_ESM.tif]
