# Supplementary material for: Elafin inhibits obesity, hyperglycemia, and liver steatosis in high-fat diet-treated male mice
Source: Sci Rep. 2020 Jul 30;10:12785. doi: 10.1038/s41598-020-69634-3 (PMC7393145; doi:10.1038/s41598-020-69634-3)
Supplement: Supplementary file 12 — Supplementary Legends. [file 41598_2020_69634_MOESM12_ESM.pdf]

Elafin inhibits obesity, hyperglycemia, and liver steatosis in high-fat diet-treated male mice.

Jiani Wang, M.D. Ph.D. [1,3]	associate professor	Emerald2015@hotmail.com
Christina Ortiz, B.S. [1]	technician	ChOrtiz@mednet.ucla.edu
Lindsey Fontenot [1],	undergraduate research student	lfontenot17@g.ucla.edu
Riya Mukhopadhyay [1],	undergraduate research student	rmuk@ucla.edu
Ying Xie, M.D. Ph.D. [1,3]	gastroenterologist	YingXie@mednet.ucla.edu
Ivy Ka Man Law, Ph.D. [1]	assistant project scientist	Klaw@mednet.ucla.edu
David Q Shih, M.D. Ph.D.[5]	gastroenterologist	David10021@gmail.com
S. Anjani Mattai, M.D.[2]	associate professor	SMattai@mednet.ucla.edu
Zhaoping Li, M.D. Ph.D.[4]	professor	Zli@mednet.ucla.edu
Hon Wai Koon, Ph.D.[1]	associate professor	hkoon@mednet.ucla.edu *

Supplementary Info File

Supplementary Table and Figure Legends:

Table S1

Baseline characteristics of patients (serum samples)

Table S2

Medication or supplement history of the patients (serum samples).

Table S3

Baseline characteristics of patients (mesenteric fat samples)

Figure S1

BMI values are not correlated with serum elafin levels in patients.

(A) BMI values in patients. (B) No strong correlation between BMI and serum elafin levels in all patient groups. (C, E, G) Serum exosomal miR181b-5p, miR210-3p, and miR219-5p expression in patients. (C) No strong correlation between serum exosomal miR181b-5p and serum elafin levels in all patient groups. (D) No strong correlation between serum exosomal miR181b-5p, miR210-3p, and miR219-5p expression in men and women without prediabetes/diabetes. (F) No strong correlation between serum exosomal miR210-3p and serum elafin levels in all patient groups. (H) No strong correlation between serum exosomal miR219-5p and serum elafin levels among women groups.

Figure S2

HFD treatment increased adipose tissue leptin and Cd36 mRNA expression in male mice.

(A) Oral glucose tolerance test. Blood glucose levels were measured at 2 hours after oral glucose (1g/kg) administration. (B) Circulating insulin, (C) total cholesterol, (D) free fatty acid, and (E) adiponectin levels in the HFD-treated or HCD-treated male mice. (F) Mesenteric fat and epididymal fat tissue leptin, adiponectin, and Cd36 mRNA expression in male mice with RD and HFD treatment. Each group consists of 8 mice.

Figure S3

Cecal microbiota was not involved in the anti-obesity effects of elafin in HFD-treated male mice.

(A) mRNA expression in mesenteric fat, epididymal fat, and subcutaneous fat of HFD-treated mice on 14 days after lentivirus injection. (B) Physiological parameters in HFD-treated mice after cecal microbiota transplantation. Each group consists of 6 mice. (C) miRNA (miR219-5p, miR210-3p, and miR219-5p) expression in mesenteric fat and epididymal fat tissues of HFD-treated mice with and without lentiviral elafin overexpression. Elafin overexpression did not affect miR219-5p, miR210-3p, and miR219-5p expression in the tested adipose tissues. (D) Serum-starved primary human mesenteric fat adipocytes (from five patients) were transiently transfected with control, miR181b-5p, or miR219-5p mimics overnight, followed by incubation with serum-free media for 6 hours. The leptin levels in the conditioned media were measured by ELISA. Each group consists of 8 mice. (E) Serum-starved mouse Raw264.7 macrophages were pretreated with fatty acid-free BSA, fatty acid-free BSA + sodium palmitate, or LPS for 2 hours, followed by incubation with elafin for 6 additional hours. Mouse TNFα levels in the conditioned media were measured by ELISA. Results are representative of two experiments.

Figure S4

Serum elafin levels were negatively correlated with serum IFNγ levels in men with T2DM.

(A) Serum cytokine levels in patients as measured by multiplex ELISA. (B) The serum cytokine levels in the mice as measured by multiplex ELISA. Each group consists of 8 mice. (C-D) Serum IFNγ levels in the men and women with T2DM. Serum IFNγ levels are negatively correlated with serum elafin levels in men with T2DM. (E) Serum IFNγ and IL-1β levels in mice. Injection of serum exosomes from elafin-overexpressing donor mice reduced serum IFNγ levels in HFD-treated mice. (F) PBMCs from healthy subjects were incubated with human serum exosomes (10μg/ml) from 12 patients without prediabetes/diabetes, 12 patients with prediabetes, and 12 patients with T2DM for 24 hours. PBMC elafin mRNA expression was significantly decreased after exposure to human serum exosomes from patients with T2DM. (G) PBMCs from healthy subjects were incubated with human serum exosomes (10μg/ml) from either 6 patients with T2DM and lower serum elafin levels or 6 patients with T2DM and high serum elafin levels for 24 hours. PBMC miR181b-5p and miR210-3p expression from high-elafin exosome-treated group was significantly higher than those from low-elafin exosome-treated group. Results are pooled from 3 independent experiments.

Figure S5

Elafin inhibits liver steatosis in HFD-treated male mice.

(A) H&E images of mouse liver tissues in regular diet-treated and HFD-treated mice with or without elafin-overexpressing lentivirus, IL-1β, or IFNγ. Liver tissues were collected 14 days after lentivirus or cytokine injection. Upper panel: 100X magnification. Lower panel: 200X magnification. Black bars represent 100μm. White lipid-containing round spaces are characteristic of hepatic steatosis. (C) H&E images of mouse liver tissues in the HFD-treated *Rag*$^{-/-}$ mice with splenocyte transplantation. (E) H&E images of mouse liver tissues in the HFD-treated mice with serum exosome transplantation and miRNA inhibitor injection. (B, D, and F) Steatosis subscore of NAFLD score of different sets of experiments. Elafin-overexpressing lentivirus or elafin-dependent splenocyte or exosome transplantation inhibited liver steatosis, which was reversed by injection of IL-1β, IFNγ, or inhibitors of miR181-5p or miR219-5p. Each group consists of 8 mice.

Figure S6

Subcutaneous or oral elafin formulation inhibits liver steatosis in HFD-treated male mice.

(A) Hepatic Cd36 mRNA expression in HFD-treated or HCD-treated mice. Lentiviral elafin overexpression inhibited liver steatosis in the HFD-treated, but not HCD-treated mice. (B) Hepatic Cd36 mRNA expression in HFD-treated mice with splenocyte transplantation. Elafin-conditioned splenocytes inhibited liver steatosis in the HFD-treated mice. (C) Hepatic Cd36 mRNA expression in HFD-treated mice with serum exosome transplantation. Elafin-conditioned serum exosomes inhibited liver steatosis in the HFD-treated mice, which was reversed by injection of miR181-5p or miR219-5p inhibitor. (D) H&E images of liver tissues in RD-treated *ob/ob* mice. Lentiviral elafin overexpression did not affect liver steatosis in the RD-treated *ob/ob* mice. (E) H&E images of liver tissues in HCD-treated WT mice. Lentiviral elafin overexpression did not affect liver steatosis in the HCD-treated mice. (F) H&E images of liver tissues in HFD-treated mice with subcutaneous PEG-elafin or oral elafin-Eudragit formulations. Both elafin-based regimens inhibited liver steatosis in the HFD-treated mice. Upper panel: 100X magnification. Lower panel: 200X magnification. Steatosis subscores of NAFLD score were shown on the right panel. (G-I) Hepatic (G) HMG-COA reductase (Hmgcoar), (H) macrophage marker (F4/80), and (I) proinflammatory cytokine (Tnf and IL-6) mRNA expression in mice. Lentiviral elafin overexpression significantly inhibited hepatic F4/80 mRNA expression in the HFD-treated, but not HCD-treated mice.

Figure S7

Serum elafin levels are not correlated with liver injury surrogate markers in men with T2DM.

(A-C) ALT, AST, ALP values of patients without prediabetes/diabetes, patients with prediabetes, and patients with T2DM. (D-F) Scatter plots show no correlations between serum elafin levels and ALT, AST, and ALP values in men and women with T2DM. (G) Comparison of clinical data of diabetic patients with or without NAFLD.

Figure S8

mRNA and miRNA expression in the mesenteric fat of non-diabetic patients.

(A) Scatter plot shows the positive correlation between leptin mRNA expression in the mesenteric fat and BMI values of non-diabetic patients. (B) Scatter plot shows no correlation between adipose elafin mRNA expression and BMI values of non-diabetic patients. (C) Scatter plots show no correlation between adipose miRNA expression and BMI values of non-diabetic patients. (D-E) Scatter plots show no correlation between adipose (miR181b-5p, miR210-3p, and miR219-5p) miRNA expression, adipose leptin mRNA expression, and adipose elafin mRNA expression in non-diabetic patients.
